# An Optimized Tissue Dissociation Protocol for Single-Cell RNA Sequencing Analysis of Fresh and Cultured Human Skin Biopsies

**DOI:** 10.3389/fcell.2022.872688

**Published:** 2022-04-28

**Authors:** Blaž Burja, Dominique Paul, Aizhan Tastanova, Sam G. Edalat, Reto Gerber, Miranda Houtman, Muriel Elhai, Kristina Bürki, Ramon Staeger, Gaetana Restivo, Ramon Lang, Snezna Sodin-Semrl, Katja Lakota, Matija Tomšič, Mitchell P. Levesque, Oliver Distler, Žiga Rotar, Mark D. Robinson, Mojca Frank-Bertoncelj

**Affiliations:** ^1^ Center of Experimental Rheumatology, Department of Rheumatology, University Hospital Zurich, University of Zurich, Zurich, Switzerland; ^2^ Department of Rheumatology, University Medical Centre Ljubljana, Ljubljana, Slovenia; ^3^ Faculty of Medicine, University of Ljubljana, Ljubljana, Slovenia; ^4^ Department of Molecular Life Sciences and Swiss Institute of Bioinformatics, University of Zurich, Zurich, Switzerland; ^5^ Department of Dermatology, University of Zurich, University Hospital Zurich, Schlieren, Switzerland; ^6^ BioMed X Institute, Heidelberg, Germany

**Keywords:** scRNAseq, skin tissue, skin biopsy, *ex vivo* explants, protocol

## Abstract

We present an optimized dissociation protocol for preparing high-quality skin cell suspensions for in-depth single-cell RNA-sequencing (scRNA-seq) analysis of fresh and cultured human skin. Our protocol enabled the isolation of a consistently high number of highly viable skin cells from small freshly dissociated punch skin biopsies, which we use for scRNA-seq studies. We recapitulated not only the main cell populations of existing single-cell skin atlases, but also identified rare cell populations, such as mast cells. Furthermore, we effectively isolated highly viable single cells from *ex vivo* cultured skin biopsy fragments and generated a global single-cell map of the explanted human skin. The quality metrics of the generated scRNA-seq datasets were comparable between freshly dissociated and cultured skin. Overall, by enabling efficient cell isolation and comprehensive cell mapping, our skin dissociation-scRNA-seq workflow can greatly facilitate scRNA-seq discoveries across diverse human skin pathologies and *ex vivo* skin explant experimentations.

## Introduction

Recent advances in single-cell RNA-seq (scRNA-seq) technology have improved our understanding of human tissue complexity in health and disease. Multiple tissue dissociation protocols have been published (Kim et al., 2020; Dubois et al., 2021), aiming at isolating viable single cells from the tissue while preserving RNA integrity and cellular tissue composition.

Careful consideration of tissue digestion protocols is crucial, especially for hard-to-digest tissues, such as skin (Jian et al., 2020), which require prolonged tissue dissociation. Various skin dissociation protocols have been utilized in the last years to study human dermal pathologies at single cell resolution. These studies revealed the previously unrecognized cell heterogeneity of the skin, including the diversity of fibroblast (Tabib et al., 2018; Solé-Boldo et al., 2020), keratinocyte (Cheng et al., 2018; Wang et al., 2020), and immune cell subtypes (He et al., 2020; Xu et al., 2021).

Variations in tissue collection, storage and processing might influence the outcomes of downstream cell analyses including scRNA-seq outputs (Denisenko et al., 2020; Slyper et al., 2020). Different stress factors, such as mechanical damage, digestion temperature (Denisenko et al., 2020; Slyper et al., 2020) and long enzymatic digestion times might skew cellular transcriptomes. Minimizing the exposure of tissue to these stress factors can be crucial for capturing representative tissue cell heterogeneity for reliable scRNA-seq results. Optimal dissociation conditions should finely balance the cell release from difficult-to-digest tissue while avoiding cellular damage during mechanical and enzymatic digestion.

Published skin dissociation protocols differ in the number of recovered cells and their viability. Additionally, the digestion times vary from 2 h to overnight digestion (Kim et al., 2020). Longer digestion times may release greater cell numbers from tissues, while negatively impacting cell viability and potentially altering original cell transcriptomes. In addition to different in-house protocols, commercial whole-skin dissociation kits, primarily optimized for fibroblast harvest (Kim et al., 2020), have often been used for skin dissociation. Notably, detailed information on tissue dissociation protocol, obtained cell yields with viability and quality control (QC) performance in scRNA-seq are rarely reported, challenging protocol standardization, study comparison, and the interpretation of results.

Small size skin biopsies are a preferred sampling strategy in routine diagnostic clinical practice, clinical translation studies and longitudinal skin sampling; however, the obtained cell yield is often low and might not be sufficient for scRNA-seq applications. Here, we describe an optimized skin digestion protocol that enables the use of small, fresh and cultured punch skin biopsies for scRNA-seq applications. Our protocol results in a high yield of viable skin cells and comprehensively captures the cell diversity of the human skin.

## Materials and Equipment

Please see Table 1.

**TABLE 1:** Key resources table.

Reagent or resource	Source	Identifier
Biological samples		
Punch skin biopsy of non-diseased skin, removed during skin surgery of patient with breast ptosis and cutaneous squamous cell carcinoma	University Hospital Zurich	N/A
Punch skin biopsies from the patients with systemic sclerosis	University Hospital Zurich	N/A
Chemicals, peptides, and recombinant proteins		
Dulbecco’s Phosphate Buffered Saline	Thermo Fisher Scientific	Cat#14190144
RPMI 1640 medium, HEPES	Thermo Fisher Scientific	Cat#52400025
Dispase II	Roche	Cat#04942078001
DNase I	Roche	Cat#11284932001
Collagenase IV	Worthington	Cat#LS004189
Fetal Bovine Serum	Corning	Cat#35-079-CV
UltraPure™ DNase/RNase-free distilled water	Thermo Fisher Scientific	Cat#10977035
UltraPure™ BSA (50 mg/ml)	Thermo Fisher Scientific	Cat#AM2616
Trypsin-EDTA (0.25%), phenol red, 100 ml	Thermo Fisher Scientific	Cat#25200056
Chromium Controller & Next GEM accessory kit	10x Genomics	Cat#1000204
Chromium Next GEM Single Cell 3′ GEM, library & gel bead kit v3.1	10x Genomics	Cat#1000121
Chromium Next GEM Chip G single cell kit	10x Genomics	Cat#1000120
Single Index Kit T Set A	10x Genomics	Cat#1000213
6-well cell culture plate	TPP	Cat#92006
Cell strainer (70 µm)	Corning	Cat#352350
ART™ wide bore filtered pipette tips	Thermo Fisher Scientific	Cat#2069G
Tissue culture dish	TPP	Cat#93100
Scalpel	B. Braun	Cat#5518067
15 ml centrifuge tubes	TPP	Cat#91015
Biopsy punch KAI 12 with plunger 20 pieces (4 mm)	Kai Medical	Cat#BPP-40F
3 piece syringes (1 ml)	BD Medical	Cat#303172
Cell strainer (40 µm)	Corning	Cat#352340
5 ml Round Bottom Polystyrene Test Tube, with Cell Strainer Snap Cap	Falcon^®^	Cat#352235
Luna Automated Cell Counter counter + Photon Slides	Logos Biosystems Inc.	Cat#L1001+ L12005
Acridine Orange/Propidium Iodide Stain	Logos Biosystems Inc.	Cat#F23001

N/A: not applicable.

### Methods

#### Patient Recruitment

We obtained control punch skin biopsies from non-diseased skin of two patients undergoing skin surgery at the Department of Dermatology, University Hospital Zurich, Switzerland. Additionally, single punch skin biopsies were collected from six patients with systemic sclerosis (SSc) who were admitted to the Department of Rheumatology, University Hospital Zurich, Switzerland for diagnostic procedures. Clinical characteristics of patients are provided in Supplementary Table S1. All patients signed the informed consent for participation in the study prior to study enrollment. The ethical committee of the Canton Zürich (ethical approvals No. EK-687, EK-800, PB-2020-00035) approved the skin collection and analysis of patients’ data and skin tissues.

#### Skin Biopsy

For each control subject, we collected two paired 4 mm punch biopsies which represented technical duplicates for tissue digest. These biopsies were placed in complete RPMI (10% FCS) medium, shipped at 4°C and freshly dissociated into a single cell suspension for scRNA-seq within 2 h of skin collection (see step by step protocol). Punch skin biopsies (4 mm) from patients with SSc (*n* = 6) were collected from nonfibrotic skin of the proximal dorsal forearm under local anesthesia with 1% Lidocaine. Biopsies were placed into complete RPMI (10% FCS) medium, shipped at 4°C, cut into small fragments with a scalpel (see step by step protocol) within 2 h of skin collection and cultured *ex vivo* for 24 h. Explanted skin fragments were then digested (*n* = 6) into single skin cell suspension (see step by step protocol) and used for scRNA-seq (*n* = 4).

#### Single-Cell RNA-seq Library Preparation and Sequencing

scRNA-seq libraries from skin cells were prepared using the single-cell 3′ v3.1 protocols and reagents (10× Genomics) according to the manufacturer’s instructions. 6,000 single skin cells were targeted for the encapsulation. The quality and quantity of cDNA and scRNA-seq libraries were assessed with Agilent Bioanalyzer (Agilent Technologies). Diluted 10 nM libraries were pooled in equimolar ratios, and the library pool was sequenced on the Illumina NovaSeq6000 sequencer (paired-end reads, R1 = 28, i7 = 8, R2 = 91, sequencing depth min. 50,000 reads/cell).

#### Bioinformatics Analysis of scRNA-seq Data From Fresh and Cultured Skin Biopsies

Cell Ranger (v2.0.2, 10× Genomics) was used to demultiplex, align the reads to Ensemble reference build GRCh38.p13 and collapse unique molecular identifiers (UMIs). The fastq files were processed into transcript count tables using Cell Ranger count (version 6.0.0) with the reference Genome GRCh38.p13 from the Genome Reference Consortium (Schneider et al., 2017). The subsequent analysis was carried out on the filtered Cell Ranger count matrix in which empty droplets had already been removed. This was done using R (version 4.1.0) and Bioconductor (version 3.13) packages. Further potential doublets were identified in the R analysis using scDblFinder (Germain et al., 2021). We ran the same processing pipeline on the fresh skin scRNA-seq data and the combined fresh and cultured scRNA-seq datasets.

The data was first filtered for cells with at least 150 expressed genes and for genes expressed in more than 10 cells. Quality control (QC) was applied based on 1) library size, 2) number of genes, 3) mitochondrial gene counts as a percentage of the library size of a cell. Cells were excluded if the library size or number of genes deviated by two median absolute deviations (MADs) below the sample median for 1) and 2), and above two MADs for 3). Furthermore, explicit filters were applied for a library size of at least 1,500 counts, a gene count of at least 500, and cells where no more than 25% of the library size was made up by mitochondrial counts. The data was normalized by library size and subsequently the top 2,000 highly variable genes were identified for dimensionality reduction. The data was batch corrected using the Harmony package (Korsunsky et al., 2019) and clustered using the Walktrap algorithm in the iGraph and bluster package (Lun, 2021). Marker gene analysis was run on the resulting clusters using Scran (Lun et al., 2016) to annotate clusters and identify cell types. As a reference for subsequent cluster annotation, cells were annotated using AUCell (Aibar et al., 2017) with skin cell markers identified by Xue et al. (2020), He et al. (2020), Tabib et al. (2021), Raynolds et al. (2021) as well as our selected marker genes for expected cell types. Cell types were then identified by annotation of the clusters. In the fresh skin dataset one of the clusters was found to contain Melanocytes and Schwann cells. Cells in this cluster were assigned to the respective cell types using a threshold of the expression of a marker gene which was one of several identified in iSEE to be capable of clearly distinguishing between the two cell types. This resulted in ten cell populations identified in the fresh samples and twelve cell populations identified in the fresh and cultured samples combined. The identified major cell populations in the fresh skin where then further subclustered and annotated for cell subtypes. For the subclustering of some cell types, the hyperparameters in the clustering method were varied to cluster cells which were identifiable as clear subgroups in feature plots.

#### Integration Analysis of Fresh Skin scRNA-seq Datasets With Published scRNA-seq Skin Atlases

We integrated our scRNA-seq data from freshly dissociated skin with skin scRNA-seq datasets from He et al. (2020), Solé-Boldo et al. (2020), and Tabib et al. (2021) using a similar pipeline as described above with some minor deviations. Only the healthy skin tissues from these datasets were included in our integration analysis. Authorized access to the data from He et al. (2020), Solé-Boldo et al. (2020), and Tabib et al. (2021) was obtained via the linked data on the Gene Expression Omnibus (GEO) accession numbers linked to the respective papers (GSE147424, GSE138669, GSE130973). As only the raw reads were available for Tabib et al., we started the data analysis with the raw Cell Ranger counts for all samples to allow a fair comparison. The four scRNA-seq datasets were subset to a list of 15,190 genes that were found to occur in at least 10 cells of each protocol and then merged to one dataset. For the QC, the MAD cut-offs for the relative filters were relaxed to 3 MADs for filters 1 to 3 (see above). Furthermore, the manually set filters for the minimum library size and gene count were lowered from 1,500 to 750 and from 750 to 500, respectively, to allow inclusion of sufficient cell numbers from all datasets. As the datasets exhibited strong heterogeneity, scTransform (Hafemeister and Satija, 2019) was chosen as a more robust normalisation method. Highly variable genes were identified by the deviance of their variance from the modelled trend using Scran (Lun et al., 2016), using a false discovery rate of 5% resulting in 1,290 genes being identified. Due to the size of the integrated dataset, the clustering method was adapted to a more efficient two-step clustering using K-means for centroid identification first and then Walktrap clustering subsequently. Ten clusters were identified which were manually annotated after analysis of the marker genes as mentioned above. Mast cells were not identified by the unsupervised clustering method but were apparent when inspecting marker genes as well as feature plots and were manually annotated based on these marker genes. Additionally, to allow for a fair comparison of detected genes across samples, we downsampled our and published datasets in a separate analysis to the sequencing depth of the dataset with the lowest coverage (Supplementary Figure S5). All datasets were downsampled using the downsampleBatches function from the scuttle R package, such that the average per-cell total count is the same across batches, i.e., in this case the samples. This analysis does not just shrink the counts by a factor across cells and samples but instead, it randomly samples counts from the data to leave all cells with a pre-specified count—in this case of the sample with the lowest coverage—thereby simulating a stronger dropout effect for samples with comparatively higher coverage.

## Results

### Step-by-Step Protocol for Skin Dissociation

**FIGURE 1:**
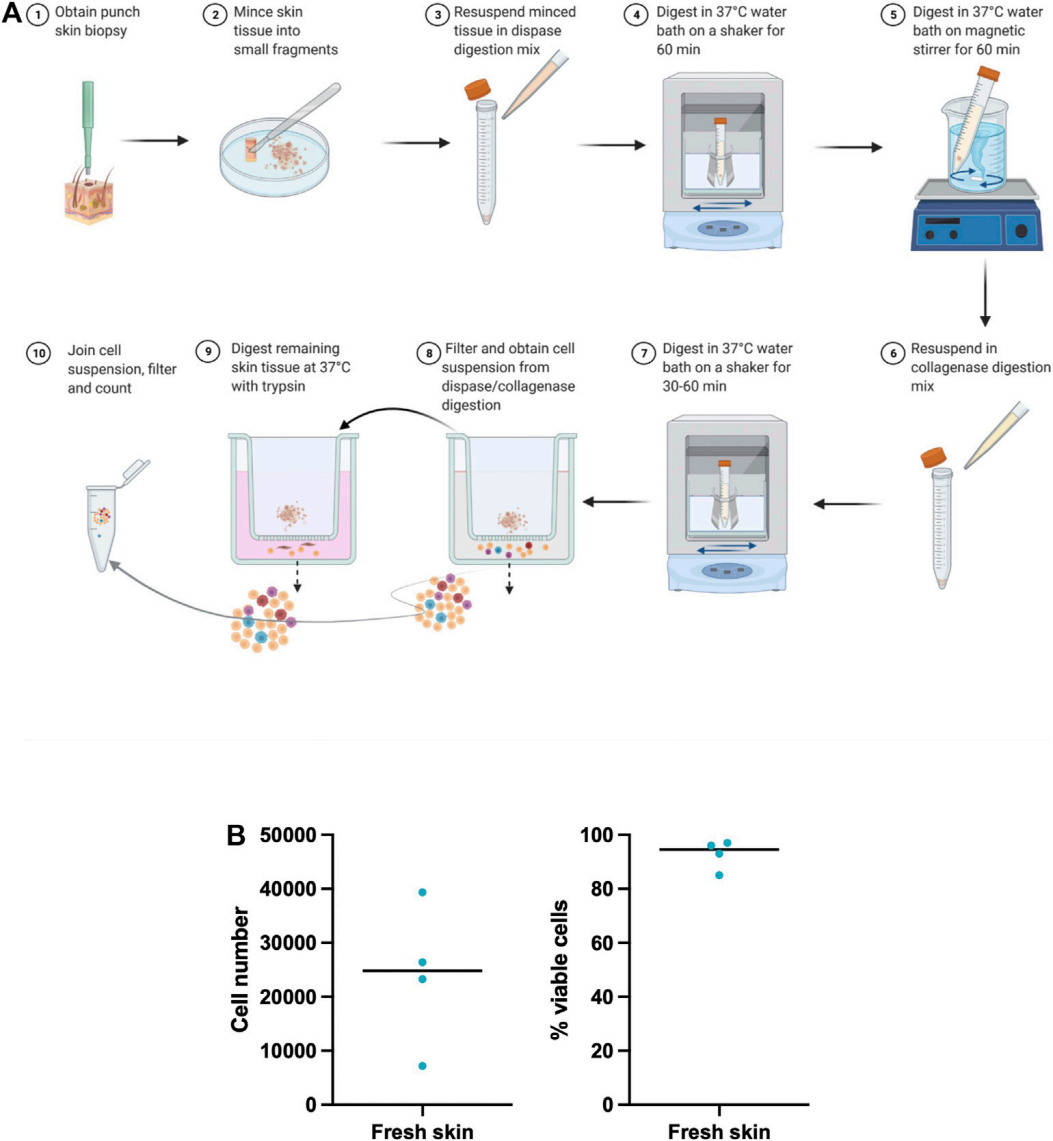
Optimized skin dissociation protocol. **(A)** Graphic representation of the principal steps of our optimized skin dissociation protocol. Figure was created with BioRender.com **(B)** Number and viability of isolated skin cells from freshly processed skin biopsies. Shown is median.

Here we describe our optimized protocol for dissociation of small sized, fresh and cultured punch biopsies from human skin. For the graphical presentation of this protocol please see Figure 1 and Supplementary Figure S1. The reagents and materials needed to conduct the protocol are described in the key resource table (Table 1).

#### Prepare Skin Tissue for Dissociation

In this step, we describe pre-processing steps to prepare fresh and cultured punch skin biopsies for subsequent tissues digestion.

Timing: 10 min

Pre-processing fresh skin

1) Start processing fresh skin, stored in complete RPMI (10% FCS) medium, within 2 h of skin biopsy acquisition.
   a) Place punch skin biopsy with sterile forceps onto a pre-wet membrane of a 70 μm cell strainer sitting in a well of a 6-well plate, filled with 4 ml RPMI (10% FCS) medium. Keep the tissue wet.
   b) Wash biopsy on the strainer three times (3×) in RPMI (10% FCS) by sequential transfer of the strainer with tissue to new wells (6-well plate) filled with 4 ml RPMI (10% FCS) medium (Supplementary Figure S1C). Always keep the tissue wet.
   • Note: Washing removes potential cell contaminants that do not originate from tissue.
   c) Transfer tissue into a RPMI (10% FCS) medium (0.5 ml) droplet placed onto a culture Petri dish (Supplementary Figure S1D). Using a sterile scalpel cut away the fat tissue.
  IMPORTANT: Complete removal of fat tissue is important, as fat droplets could interfere with subsequent enzymatic tissue digestion.
   d) Transfer skin biopsy with sterile forceps onto a pre-wet membrane of a 70 μm cell strainer placed into a well of a 6-well plate, filled with 4 ml PBS.
   e) Wash three times (3×) with PBS by sequential transfer of strainer into new wells (6-well plate) filled with 4 ml PBS. Always keep tissue wet.
   • Note: Washing removes potential remaining fat tissue contaminants.

Skin explant culture

2) To prepare skin biopsy for explant culture, proceed with skin processing within 2 h of biopsy acquision.
3) a) Transfer skin tissue onto a dish with 750 μl droplet of RPMI (10% FCS) medium and cut with a scalpel within the droplet (1 punch biopsy to be cut into around 10 pieces of approximate 1 mm × 1.2 mm size, Supplementary Figure S1E).
   b) Transfer tissue fragments into cell culture well with defined treatment conditions for 24 h.
   c) After 24 h, transfer tissue fragments directly into dispase digestion mix (see step 2 of the protocol), and inspect the wells under the microscope for any remaining attached cells. Collect the culture supernatants for downstream analyses, if applicable.
  Note: In our experience cells did not migrate out of the tissue fragments during the 24 h culture. In case any remaining cells exist, attached or floating, these cells can be collected by trypsinization and supernatant centrifugation, respectively, the wells additionally washed with RPMI (10% FCS) and cells added to the tissue fragments in dispase digestion mix.

#### Dissociation of Skin With Dispase

In this step, we dissociate fresh or cultured skin tissue fragments into a suspension of single cells and partially digested tissue fragments using the combined enzymatic-mechanical tissue dissociation protocol involving dispase II. Dispase is a protease that hydrolyzes the N-terminal peptide bonds of non-polar amino acid residues (Volovitz et al., 2016). It cleaves extracellular matrix components such as fibronectin, collagen IV and to a lesser extent collagen I and is thus used to gently dissociate skin tissue.

Timing: 150 min

1) Prepare dispase digestion mix in a 15 ml conical tube.
  a) To prepare the dispase digestion mix for one punch biopsy (4 mm) mix: 3 ml D-PBS with 0.75 ml of 9.6 U/ml Dispase II, 30 μl of 50 mM CaCl_2_, 90 μl of 1% BSA and 30 μl of 10 mg/ml DNAse I. The final working dispase concentration in this mix is then 2.4 U/ml.
  b) Prewarm dispase digestion mix in a water bath at 37°C*.*
  c) Transfer 500 μl dispase digestion mix drop onto the culture Petri dish for tissue processing and 2 ml of mix into a new 15 ml falcon tube for digest.
2) Mince skin tissue.
  a) Transfer fresh washed punch skin biopsies or cultured skin fragments with a forceps into the dispase digestion mix drop on a Petri dish. If tissue fragments stick to the forceps, remove them gently by combined use of a forceps and a scalpel.
  b) Gently mince skin tissue within the drop with a sterile scalpel into tiny tissue fragments (∼0.5 mm), while keeping tissue wet (Supplementary Figure S1G). Transfer 500 μl dispase digestion mix with minced tissue fragments into the 15 ml falcon tube with dispase digestion mix (2 ml).
  • Note: Cut skin gently by scalpel to avoid extensively damaging plastic petri dish surface and contaminating tissue fragments with plastic particles. This might later increase the probability of clogging the 10x Genomics chips during GEM droplet formation. Alternatively, a sterile glass surface can be used to mince the skin tissue.
3) Digest skin tissue at 37°C using a mixed enzymatic-mechanical protocol with dispase.
   a) To start tissue digestion, place the tube with tissue fragments into a rack within a 37°C water bath-like system on a pre-warmed shaker chamber (37°C) (Supplementary Figure S1J). Digest tissue for 60 min at 37°C with continuous shaking at 100 rpm.
  CRITICAL: The water bath helps keeping the reaction temperature at 37°C during tissue digestion. Measure the water bath’s temperature every 20 min and replace with warm water, e.g., to keep the temperature at 37°C.
   b) In the next step, add sterile magnetic stirrers to tissue digestion tube under sterile conditions and close the tube safely. Transfer the tube into a beaker containing pre-warmed water (37°C) on a magnetic holder within the pre-warmed oven (37°C) (Supplementary Figure S1K).
   c) Incubate the tissue at 37°C in the enzymatic mixture for additional 60 min with a continuous magnetic stirring.
  Note: A combination of a ball-shaped and a cylinder-shaped magnetic stirrers facilitates tissue mixing during digestion. Stirring speed should keep the tissue solution in a steady movement, however too fast stirring could facilitate shear forces that could damage the cell.
4) At the end of stirring time, collect magnetic stirrers with a sterile magnetic stick, and inspect the stirrers for any sticking tissue fragments under sterile conditions. Avoid losing precious tissue fragments that stick to the stirrers by returning them into tissue digestion mix.
5) Centrifuge reaction mix with dispase-digested tissue at 300 × g, room temperature for 10 min, discard the supernatant, and transfer the pellet in the next dissociation step.

#### Dissociation of Skin Tissue—Collagenase IV

In this step, dispase pre-digested skin tissue fragments are further dissociated into a suspension of single cells using the combined enzymatic-mechanical tissue dissociation protocol which includes collagenase IV. Collagenase IV is designed to be especially low in tryptic activity to limit damage to membrane proteins and receptors. It cleaves the bond between a neutral amino acid (X) and glycin, abundant in collagen, thus it is able to break the peptide bonds in collagen, facilitating extracellular matrix digestion and releasing cells into suspension (Seltzer et al., 1990).

Timing: 80 min

1) Prepare collagenase digestion mix in a conical 15 ml polystyrene tube.
  a) To prepare collagenase digestion mix for one punch biopsy (4 mm) combine 3 ml of PBS with Collagenase IV, 30 μl of 100 mM CaCl_2_, 90 μl of 1% BSA and 30 μl of 10 mg/ml DNAse I. The final working collagenase IV concentration in this mix should be 1000 U/ml.
  b) Prewarm collagenase digestion mix in a water bath at 37°C.
2) Digest skin tissue at 37°C in a mixed enzymatic-mechanical protocol.
  a) Add 2.5 ml of collagenase digestion mix to loosen pellet obtained from the dispase dissociation step and resuspend the cells and tissue fragments.
  b) Place the falcon tube into a rack within the 37°C water bath-like system (Supplementary Figure S1J) on a pre-warmed shaker chamber (37°C). Digest for 30 min with a continuous tube shaking at 100 rpm within the chamber (37°C).
3) Filter cell suspension after completed collagenase digestion and enrich for tissue retained cells.
  a) Pre-wet the 70 μm strainer with washing buffer (0.2% BSA in PBS) in the 6-well plate.
  b) Pipette the collagenase reaction mix with dissociated cells and remaining tissue fragments using a 1 ml wide-bore pipet tip through the pre-wet 70 μm cell strainer into a well of a 6-well plate, containing 1 ml of wash buffer (0.2% BSA in PBS). Large tissue debris, undigested tissue pieces, and cell clumps will remain on the strainer.
  c) Using the syringe plunger head, gently press the skin tissue fragments against the strainer’s bottom into the wash buffer (0.2% BSA in PBS) to facilitate remaining cell release from the tissue fragments. Collect any remaining drops of cell suspension from the bottom side of the strainer with a fresh pipette tip and return them to the filtered cell suspension in the well.
  • Note: Undigested tissue left on the cell strainer is in parallel digested with trypsin (see the trypsin digestion step of the protocol).
  d) Collect cell suspension and filter it through 40 μm strainer into a fresh falcon tube. Wash the well twice with 4.5 ml of RPMI (10% FCS) to collect any remaining cells and add to this medium to the cell suspension by filtering it through the 40 μm strainer.
  • Note: 10% FCS RPMI stops the enzymatic digestion process.
4) Centrifuge the cell suspension at 300xg, room temperature for 10 min. Remove the supernatant. Gently flick the pellet and resuspend the cells with wide-bore tips in 50 μl of 0.2% BSA PBS. Keep cells on ice.

#### Maximizing Cell Yield by Trypsin Digestion of Remaining Tissue Fragments

In this step, the remaining tissue fragments are additionally digested with trypsin, to release any remaining cells and maximize cell yield. The tissue debris and cell aggregates are removed. In our experience this step contributed to further cell enrichment increasing cell counts from 16% to 44% with median cell viability of 88%.

Timing: 30 min

1) Pre-warm 3 ml/well of 0.25% trypsin in a 6-well plate kept in cell culture incubator at 37°C.
2) Trypsinize the remaining skin tissue fragments.
  a) Transfer the strainers with undigested tissue pieces from the step 3c. of collagenase digestion into wells with pre-warmed trypsin and trypsinize for 15 min at 37°C. Gently pipette the reaction mix with tissue pieces 5× up and down through the 70 μm cell strainer using a wide-bore pipet tip; repeat 3× during the trypsinization process.
  b) Filter the cell suspension through 40 μm strainer into a new 15 ml falcon tube. Discard tissue remnants on the strainer.
  c) Stop trypsinization process with 10% FCS RPMI. Specifically, wash the wells with 2 × 5 ml 10% FCS RPMI and add it through 40 μm strainer to the filtered cell suspension.
3) Centrifuge the cell suspension at 300 × g, room temperature for 10 min, discard the supernatant, gently flick the pellet and resuspend the cells with wide-bore tips in 20 μl 0.2% BSA PBS. Keep the cells on ice.
4) Combine cell suspensions from dispase/collagenase and trypsin digestion steps.

#### Prepare Single-Cell Suspension for Single-Cell RNA Sequencing (10× Genomics)

In this step, cell yield and viability are determined, potential cell debris and aggregates removed by additional filtering, and cell suspension are diluted or concentrated to be ready for starting the Gel Bead-in-Emulsion (GEM) generation.

Timing: 15 min

1) Filter the combined single cell suspension through the 35 μm strainer cap of Falcon 5 ml tubes (Catalog number #100-0087) into a 1.5 ml Eppendorf tube, repeat filtering two times.
  • CRITICAL: This step prevents clogging of the microchannels on the 10× Genomics Next GEM Chip G.
2) Determine cell number and viability, for example, by using acridine orange (AO) propidium iodide stain (PI) for live/dead cells according to manufacturer’s guidelines and count cells with Luna cell counter (Logos Biosystms, Luna settings: dilution factor 2, cell size gating 3–90 μm).
  • Note: Luna measurements enable also the inspection of cell suspensions for remaining debris and cell aggregates in bright field. In case of remaining debris or cell clumps, repeat filtering the cell suspension through the 35 μm strainer.
3) Adjust the cell number to final concentration of (700–1,200 skin cells/μl) in accordance with the 10× Genomics protocol guidelines. For diluting the cell suspension use 0.2% BSA D-PBS.
  CRITICAL: In our experience, the concentration of 700 cells/μl minimizes the probability of chip clogging.
4) Keep the cells on ice until starting the 10× Genomics protocol for single-cell droplet generation (GEM).

### Optimized Skin Dissociation Protocol Results in a High Yield and Viability of Isolated Cells From Fresh Skin

A skin digestion protocol was used for the dissociation of cells from paired fresh skin punch biopsies from two donors. We isolated 24,053 total skin cells per a 4 mm punch biopsy with average cell viability of 92.75% (Figure 1B) pointing to a good cell yield and survival. We utilized two fresh punch biopsies for scRNA-seq analysis and generation of fresh skin cell atlases. By comparing cell yields and survival across our and published (Supplementary Table S2) skin dissociation protocols, we noted a higher number and viability of isolated skin cells in our protocol. However, a direct comparison of these protocols on paired skin biopsies from the same donor is needed to fully confirm this observation.

#### A Single-Cell Transcriptomic Atlas of Fresh Human Skin Cells Efficiently Captures Cellular Skin Composition and Identifies Rare Skin Cell Types

**FIGURE 2:**
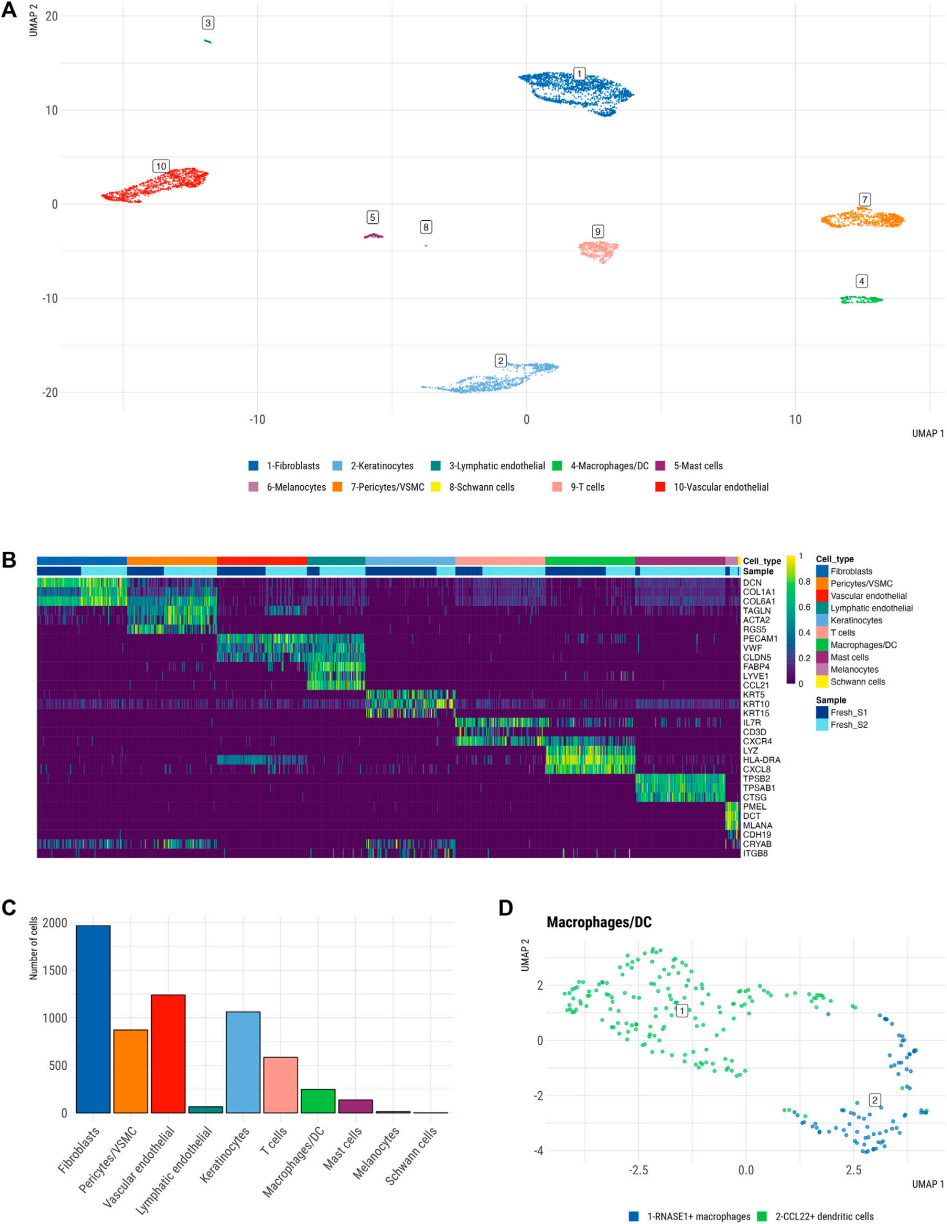
Single cell atlas of freshly dissociated human skin. **(A)** Uniform manifold approximation and projection (UMAP) plot depicting single-cell transcriptomes from whole human skin (*n* = 2), coloured by cell type. Each dot represents a single cell (*n* = 6,192). Color-code defined by manual cluster annotation after unsupervised clustering was performed via Walktrap clustering (see *Methods*). Shown are 10 principal skin cell populations including fibroblasts, keratinocytes, lymphatic endothelial cells, macrophages/DC, mast cells, melanocytes, pericytes/VSMCs, Schwann cells, T cells, vascular endothelial. **(B)** Heatmap showing the three marker genes of each cell cluster. Each column represents a single cell, each row represents an individual gene. A maximum of a hundred cells were sampled per cell cluster. Magnitude of gene expression measured in scaled log normalized UMI counts. Yellow indicates maximum gene expression and purple indicates no expression of the gene. **(C)** Relative abundance of main cell types across integrated fresh skin samples. **(D)** Macrophage/DC cluster containing a larger population of 165 DCs and a smaller population of 82 macrophages.

To generate a single cell atlas of the fresh human skin, we analyzed scRNA-seq data from freshly dissociated skin samples. Specifically, 6,192 freshly dissociated cells passed QC (see *Methods*). Detailed QC analysis of scRNA-seq datasets from freshly dissociated samples showed a similar distribution of library sizes and identified genes per cell with average of 2,767 and 2,538 genes detected per sample, respectively. Furthermore, both samples exhibited a sufficiently high number of viable cells and did not exhibit any aberrant behavior. Filtered high quality cells, that passed QC, segregated into ten major clusters (Figure 2A), which displayed distinct expression profile of cell marker genes (Figure 2B). These cell clusters represented ten main cell types including COL1A1+ DCN+ fibroblasts, KRT5+/KRT10+ keratinocytes, TAGLN+ pericytes/vascular smooth muscle cells (VSMCs), PECAM1+ vascular endothelial cells, LYVE1+ lymphatic endothelial cells, LYZ+ macrophages/dendritic cells (DCs), CXCR4+ T cells, TPSB2+ mast cells, PMEL+ melanocytes and CDH19+ Schwann cells (Figures 2B,C). Macrophages (cluster 1, Figure 2D) and DC (cluster 2, Figure 2D) were distinguished by elevated expression of previously described markers of dermal macrophages (*CD14*, *CD68*, *CD163*) (Fuentes-Duculan et al., 2010), while DCs expressed *CCR7* and *CCL22*, which are markers of mature DCs.

#### Subclustering Analysis Unravels the Complex Composition of Large Skin Cell Populations

Fibroblasts, keratinocytes and pericytes/VSMC were among the largest resident skin cell populations (Figure 2C) and were further subclustered to identify their subsets.

**FIGURE 3:**
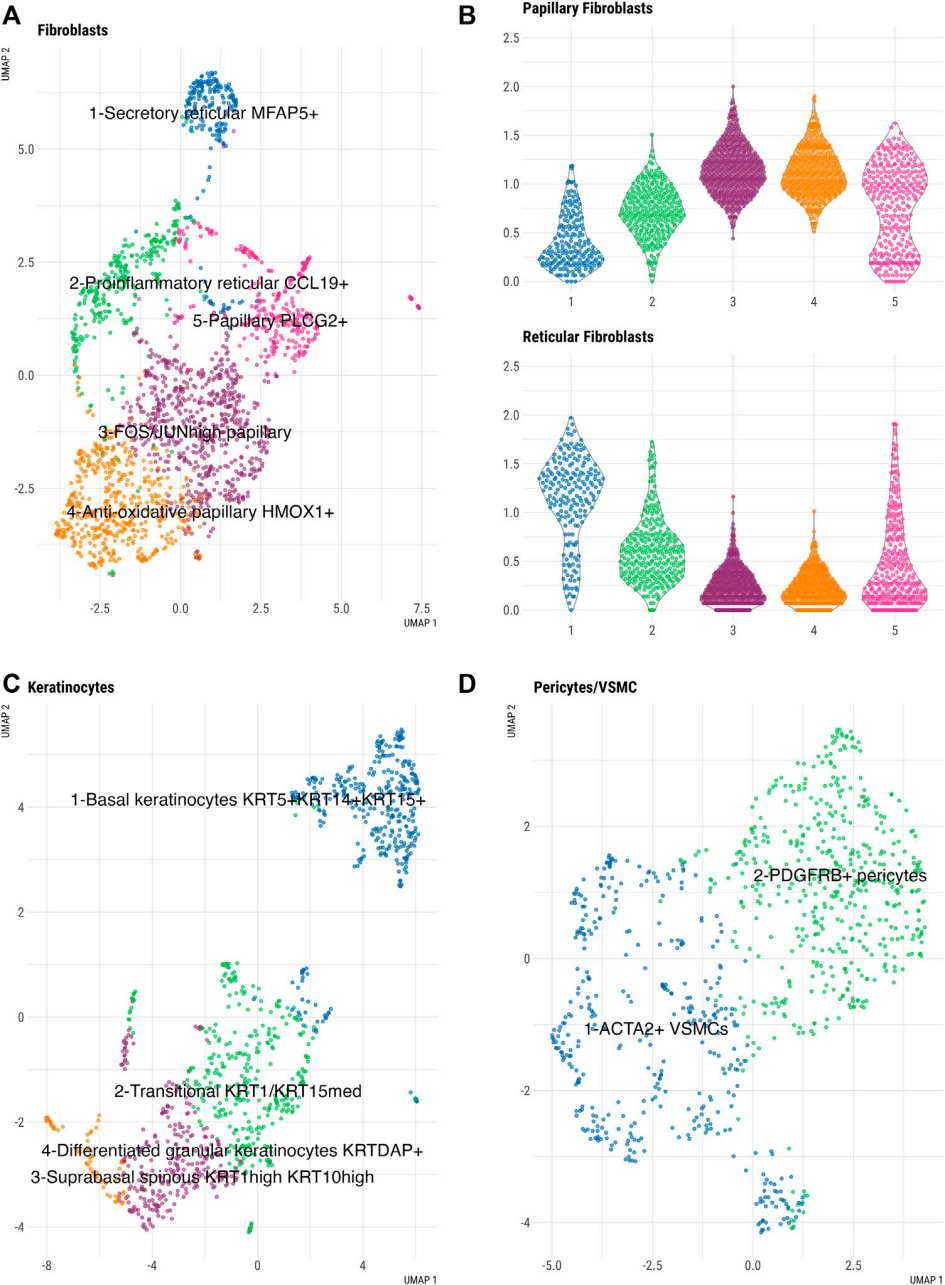
Subclustering analysis of large skin cell populations in freshly dissociated skin. **(A)** UMAP of fibroblast subclusters **(B)** Violin plots of averaged expression of the genes constituting the papillary (top panel) and reticular (bottom panel) gene signatures (See Supplementary Figure S5A for the list of genes) across the five fibroblast clusters. x-axis depicts cell cluster number and y-axis represents average expression of each set of genes using log-normalized UMI counts. **(C)** UMAP of keratinocyte subclusters and **(D)** UMAP of pericytes/VSMCs subclusters.

By subclustering the fibroblasts in our fresh skin cell atlas, we identified five distinct fibroblast subpopulations (Figure 3A, Supplementary Figure S2A). Dermal fibroblasts show large diversity; for example, different body sites and skin compartments, such as papillary and reticular dermis, are populated by transcriptionally and functionally specialized fibroblast subsets (Janson et al., 2012). To determine whether our five fibroblast clusters represent spatially-specialized dermal fibroblast populations, we analyzed the expression of known markers of papillary and reticular dermal fibroblasts across clusters (Supplementary Figure S3). A papillary gene expression signature (Figure 3B) was mostly restricted to fibroblast clusters 3, 4, and 5, with the highest expression in clusters 3 (FOS+ JUN+ fibroblasts) and 4 (HMOX1+ TNFAIP6+ fibroblasts). In contrast, cluster 1 showed a prominent reticular gene signature, including *MGP*, *ANGTPL1*, *FGF7*, *EFEMP1*, *MFAP5*, suggesting that these cells represent secretory reticular dermal fibroblasts. Therefore, our subclustering analysis confirmed the presence of main spatially-specialized fibroblast subtypes with an additional distinct fibroblast cluster (cluster 2), defined by a mixed papillary/-reticular signature and a prominent inflammatory gene expression signature (*CCL19, C3*) (Figure 3A). Looking closer in papillary dermal fibroblast subsets, we uncovered enriched transcriptional regulation signature in cluster 3 and anti-oxidative/immune-regulatory gene signature in fibroblast cluster 4. Specifically, cluster 4 fibroblasts expressed genes involved in anti-oxidative response (*HMOX1*, *SOD2*, *FTH*, *SQSTM*) and immunomodulatory (*SQSTM1*, *TNFAIP6*, *TNFAIP3*, *NFKB1*) cell functions, while cluster 3 fibroblasts (FOS/JUN^high^) resembled pro-inflammatory cluster 2 fibroblasts, but with higher collagen gene expression, confirming their papillary characteristics.

Further, we identified four distinct keratinocyte clusters that corresponded to keratinocyte differentiation states and epidermal location (Figure 3C, Supplementary Figure S2B). Keratinocytes in cluster 1 were enriched for known basal keratinocyte markers (*KRT5*, *KRT14*, *KRT15*), while cluster 3 keratinocytes, characterized by *KRT1* and *KRT10* expression, showed a gene signature of suprabasal spinous keratinocytes. *KRT1* and *KRT10* were also expressed in cluster 4 cells with a signature of differentiated granular keratinocytes (KRTDAP+). An additional keratinocyte population, which clustered separately (cluster 2), represented a transitional state between basal and suprabasal keratinocytes. A differentiated granular keratinocyte cluster (cluster 4) expressed the known differentiation gene markers (*KRT2*, *IVL*, *SBSN*, *CALML5*, *DSC1*) and contained a small subset of LOR+ FLG*+* cells which could represent terminally differentiated cells of the cornified envelope (Hughes et al., 2020).

Pericyte/VSMCs (Figure 3D, Supplementary Figure S2C) separated into two different clusters. Based on the expression of established lineage markers, we identified one cluster of VMSC expressing *ACTA2*, *TAGLN*, *MYH11*, *CNN1*, *TPM2* cells and one cluster of *PDGFRB+* pericytes.

### Skin scRNA-Seq Data Generated by Our Protocol Exhibit Comparable Quality Control Metrices and Skin Composition to Published Human Skin Datasets

**FIGURE 4:**
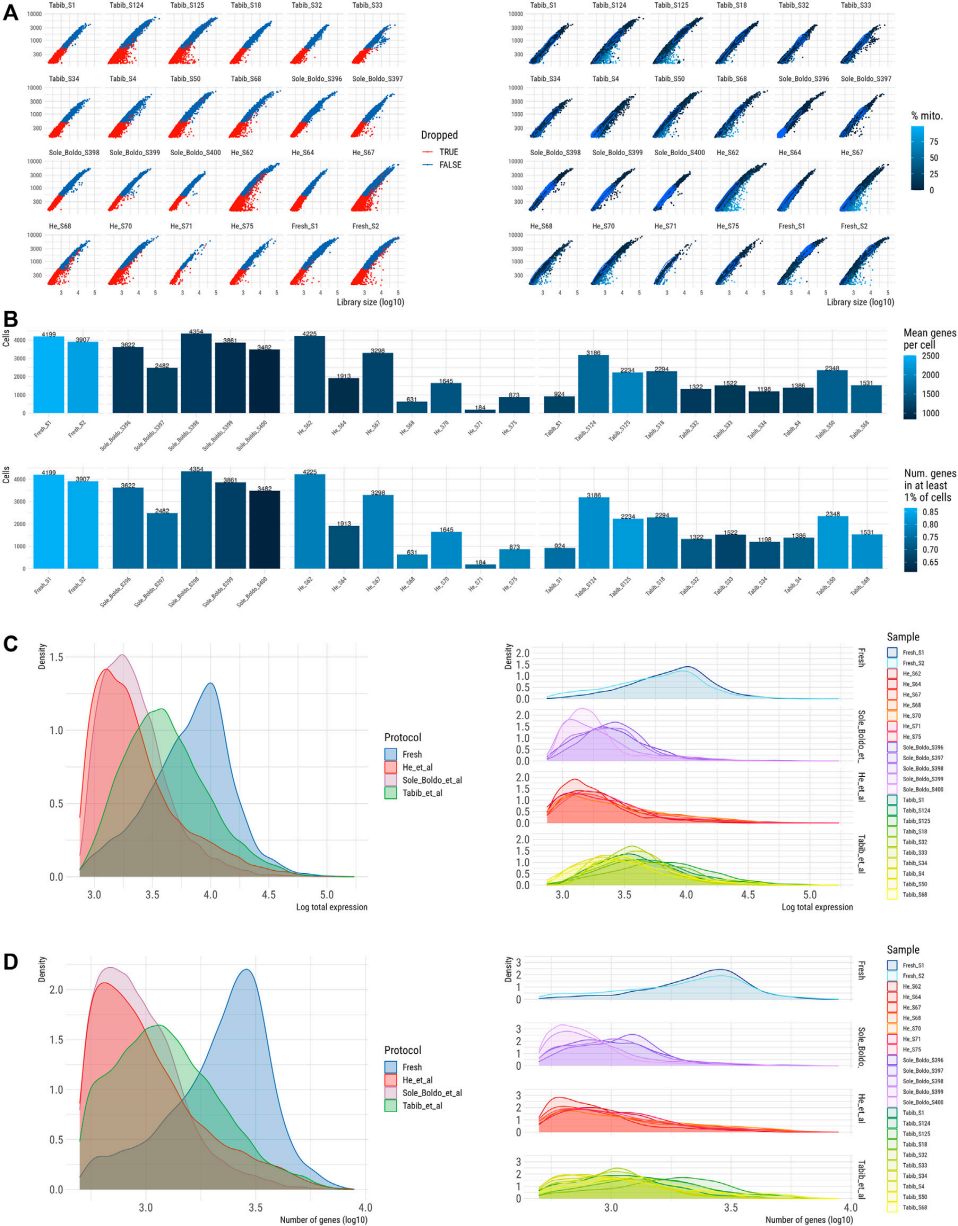
QC of skin scRNA-seq dataset prior to normalisation and batch correction **(A)**. Library size (x-axis) vs. number of genes per sample coloured by filter (left) and percentage of mitochondrial counts (right). **(B)** Number of cells per sample coloured by number of genes (top) and by number of genes in at least 1% of cells (bottom). Distribution of counts **(C)** per protocol (left) and per sample (right). Distribution of number of genes **(D)** per protocol (left) and per sample (right).

To evaluate the performance of our fresh skin scRNA-seq data generated by the optimized skin digestion protocol in terms of QC metrics and recapitulation of skin cell populations, we integrated our data with publicly available scRNA-seq datasets from healthy skin. These datasets were created from freshly dissociated skin except for He et al. who used cryopreserved skin samples, while dissociation protocols varied across the studies (Supplementary Table S3). We excluded low quality cells based on the manually set filters for gene count and library size (see *Methods*). This QC approach filtered out low quality cells from all samples to a similar degree (Supplementary Figure S4A). The datasets from Tabib et al. as well as ours had a comparable ratio of mitochondrial reads, which was lowest in the study of Solé-Boldo et al. and highest in the study by He et al. (Figure 4A). These findings could reflect the inclusion of the dead cell removal step in the Solé-Boldo skin dissociation protocol and cryopreservation of skin in He et al., which might be associated with decreased cell survival (Supplementary Table S3).

In total, 56,621 high quality single skin cells passed QC (Figure 4B) including 8,106 cells from our study, 17,945 cells from Tabib et al. (2021), 12,769 cells from He et al. (2020) and 17,801 healthy skin cells from Solé-Boldo et al. (2020). Notably, the number of cells varied significantly between the seven samples in the He et al. dataset and three samples had less than 1,000 cells passing QC (Figure 4B). Compared to other datasets, our dataset demonstrated 1.9–3.9 higher values for median library size per cell and 2-2.7 times higher median number of detected genes. (Figures 4C,D, Supplementary Figure S4B). These differences could arise due to a higher sequencing depth used in our scRNA-seq protocol. Thus, we downsampled the reads in our and published datasets to the sequencing depth of a sample with the lowest coverage. This analysis demonstrated a comparable number of the detected genes across all the samples (Supplementary Figure S5), further confirming that the observed differences in the number of detected genes across the samples could be attributed to the differences in sequencing depths.

**FIGURE 5:**
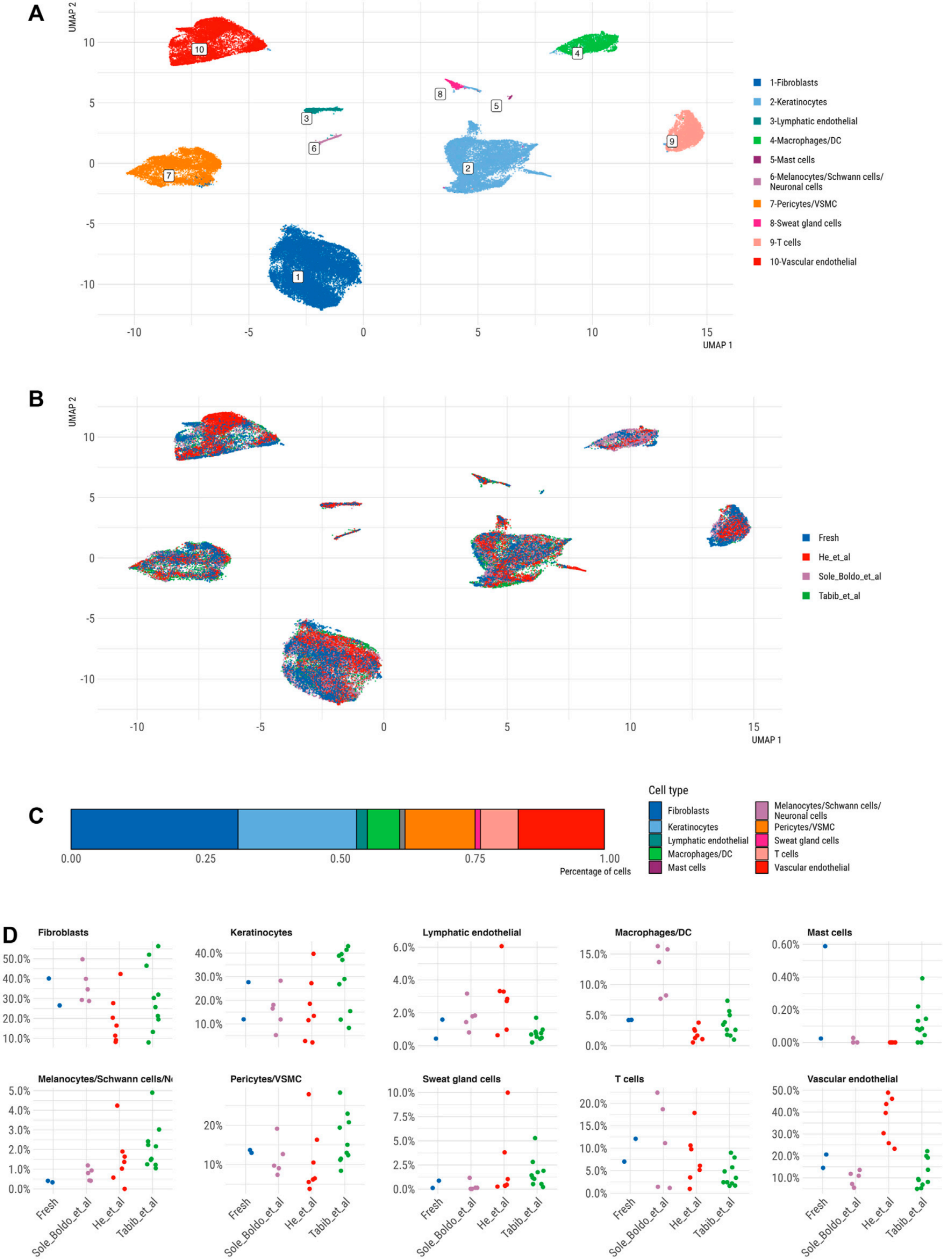
UMAP representation of integrated skin scRNA-seq datasets, coloured by **(A)** main cell type and **(B)** by dataset. **(C)** Bar plot showing relative abundance of cell type distribution across integrated dataset **(D)** Jitter strip plot showing relative abundance of cell types across all differerent datasets.

After successful QC analysis, we integrated our and published datasets to generate a large skin cell atlas and determine how our dataset performs compared to other published human scRNA-seq studies. The UMAP and clustering of the integrated scRNA-seq skin cell profiles revealed ten distinct clusters corresponding to principal skin populations (Figures 5A,B). Specifically, these populations included DCN+ fibroblasts, TAGLN+ pericyte/VSMC, PECAM1+ vascular ECs, CCL21+ lymphatic ECs, SFN+ keratinocytes, CXCL8+/HLA-DRA+ macrophages/DC, CD52+ T cells, TPSAB1+ mast cells, AQP5+ sweat gland cells and a combined cluster of MLANA+ melanocytes, CRYAB+ Schwann cells and GPM6B+ neuronal cells (Figure 5A). Fibroblasts represented the most abundant cell type (31%), followed by keratinocytes (22%), vascular endothelial cells (17%), pericytes/VSMC (13%), T cells (7%) and macrophages/DCs (6%); mast cells (0.1%), lymphatic endothelial cells (2%), sweat gland cells (1%) and melanocytes/Schwann cells/neuronal cells (1%) represented minor skin cell populations (Figure 5C).

While every dataset contributed cells to the majority of clusters (Figure 5B), certain datasets appeared slightly enriched in specific cell types. Comparing cell population abundances across the datasets demonstrated a consistently higher proportion of vascular ECs in the He et al. dataset, of macrophages/DCs in the Solé-Boldo study and of melanocytes/Schwann/neuronal cells in the Tabib et al. data (Figure 5D). The differential abundance analysis demonstrated a significant enrichment of melanocytes/Schwann cells/neuronal cells in Tabib et al. compared to our data, while other cell types were comparably represented across both datasets (Supplementary Figure S6). Yet, these results must be taken by caution given the low power of our dataset containing only two skin samples.

Our dataset efficiently captured all skin cell populations including mast cells (Figure 5D). Mast cells were present in fresh skin datasets, but absent from all cryopreserved skin samples in the He et al. study, indicating their potential loss due to cryopreservation. One of our samples showed the highest proportion of detected mast cells across published skin scRNA-seq, but further studies on larger patient cohorts are needed to understand the factors (e.g., skin location) influencing mast cell abundances in scRNA-seq datasets.

### The Optimized Digestion Protocol Facilitates the Generation of High Quality scRNA-Seq Data From Explanted Skin Biopsies

Cultured skin explants represent experimental *ex vivo* skin models for research and drug target discovery in human skin pathologies. Utilizing scRNA-seq as a readout in such experiments can deconvolute drug effects across skin cell populations, thereby providing valuable insight into cell type-specific but also tissue cell community-based drug actions. Therefore, we explored whether our dissociation protocol can facilitate efficient cell isolation and scRNA-seq analysis also on cultured skin explants from clinically relevant patient material. Our cultured skin explants were derived from patients with SSc as a prototype of fibrotic skin pathology. We then compared the quality metrics of scRNA-seq datasets generated from cultured versus fresh human skin utilizing our fresh skin datasets from subjects undergoing skin surgery.

In total, we dissociated cultured skin explants from six SSc donors and isolated on average 18,535 skin cells from a 4 mm punch biopsy with average cell viability of 91.22% yielding comparable results to the freshly dissociated skin (Supplementary Figure S7). We utilized cultured explant samples from four SSc patients for scRNA-seq data generation and analysis.

We compared the quality of scRNA-seq data generated from cultured and freshly dissociated skin. In total, 17,837 cells passed QC (see *Methods*). Cells were filtered out in combination of all filters, including 1) library size 2) number of genes and 3) mitochondrial gene counts as percentage of the library size; no single filter was responsible for the exclusion of most cells. All cultured and fresh datasets, except Culture_S1 data, had a similar distribution of library sizes per cell and genes identified per cell (Supplementary Figures S8A,B). Compared to other samples, Culture_S1 had a significantly lower number of sequenced cells, and only 418 cells (42.8%) were kept after QC making it the only sample with rather few cells (Supplementary Figure S8B). While this sample demonstrated bimodal distribution of cell library size (Supplementary Figure S8C) and gene counts (Supplementary Figure S8D), other samples did not differ in these QC metrics (Supplementary Figures S8C,D) and were excluded to a similar degree (Supplementary Figure S9A). Thus, our skin digestion protocol enabled efficient cell isolation from *ex vivo* explanted skin tissue for scRNA-studies. Short term skin culture (24 h) did not significantly alter single-cell’s quality metrics compared to fresh skin, suggesting that our culturing conditions were suitable for scRNA-seq studies on clinically relevant cultured human skin explants.

**FIGURE 6:**
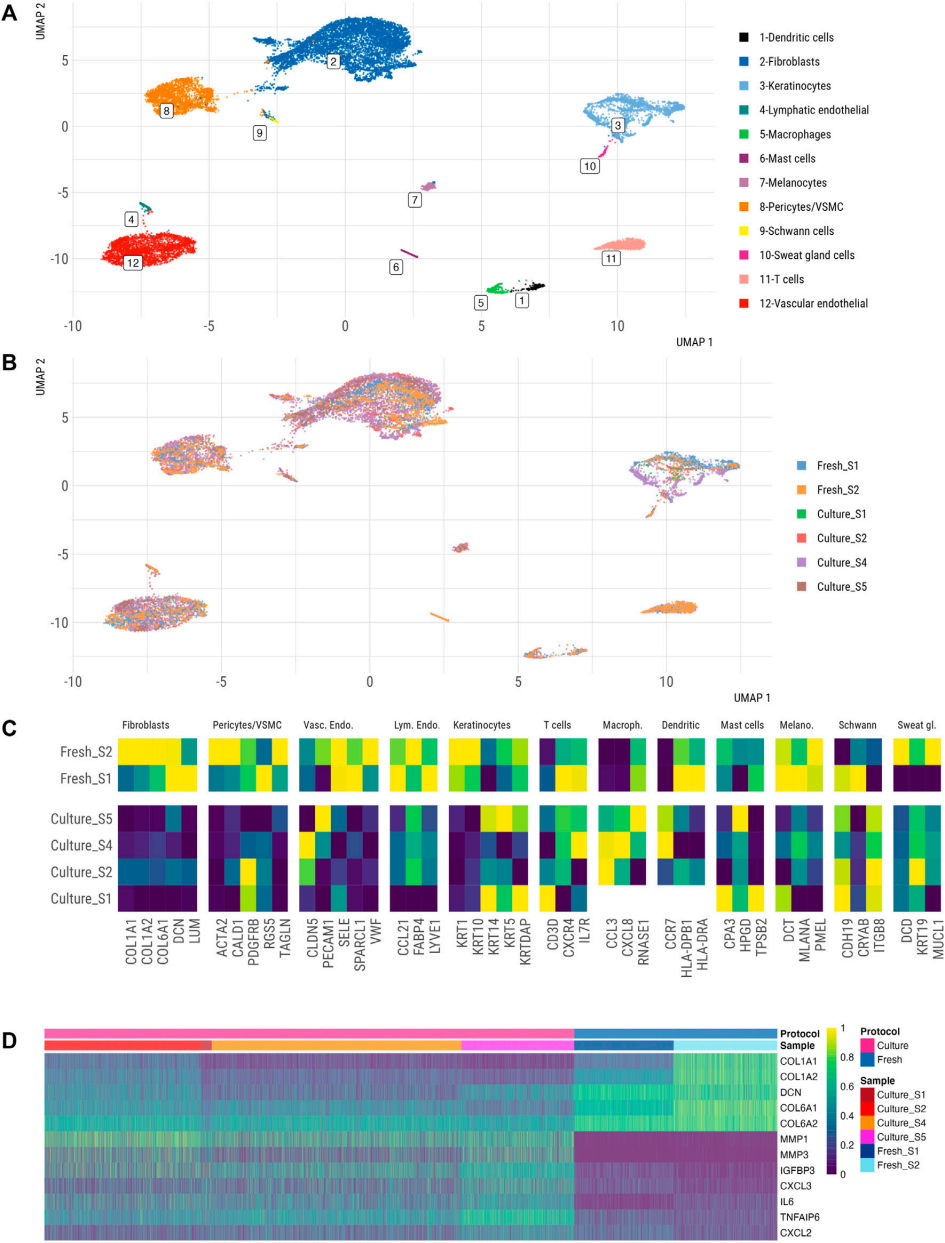
Single cell atlas of freshly vs. *ex-vivo* dissociated human skin. UMAP representation of fresh and cultured skin samples, coloured by annotated cell type **(A)** and by **(B)** sample type (fresh vs. cultured). **(C)** Marker gene expression of 3–5 markers of each cluster from healthy vs. cultured skin datasets. Averaged expression of markers within the identified cell type is shown. Marker genes were identified in an unbiased fashion blind to known cell type markers. **(D)** Heatmap of fibroblasts’ gene expression from fresh vs. cultured samples.

Next, we integrated the filtered scRNA-seq datasets from fresh and cultured skin to gain first insights into cellular composition and transcriptomes of cultured skin explants. We identified twelve distinct cell clusters in fresh skin and cultured skin explants, corresponding to DCN+ fibroblasts, SFN+ keratinocytes, PECAM1+ vascular ECs, FABP4+ lymphatic ECs, CALD1+ pericytes/VSMCs, CXCR4+ T cells, CCL3+ macrophages, CCR7+ dendritic cells, TPSB2+ mast cells, PMEL+ melanocytes, CDH19+ Schwann cells and MUCL1+ sweat gland cells (Figure 6A). Notably, cultured skin explants contained all main cell populations of freshly dissociated skin.

We further explored the transcriptomes of single cells within major skin cell populations in cultured and fresh skin datasets. We observed significant alterations in the expression of several cell identity markers in the cultured skin dataset across different cell populations (Figure 6C). These changes were especially evident in fibroblasts (*COL1A1*, *COL1A2*, *COL6A1*, *DCN*, *LUM*), pericytes/VSMCs (*ACTA2*, *CALD1*, *RGS5*, *TAGLN*), keratinocytes (*KRT1*, *KRT10*, *KRTDAP*), vascular endothelial cells (*SELE*, *SPARCL1*, *VWF*) and lymphatic endothelial cells (*CCL21*, *FABP4*, *LYVE1*), while absent from immune cells (T cells, macrophages, dendritic cells) and neural crest cells (melanocytes, Schwann cells) (Figure 6C). Overall, fibroblasts showed the largest gene expression alterations (Figures 6B,D); differential state analysis revealed decreased expression of extracellular matrix (ECM)-related genes (*COL1A1*, *COL1A2*, *DCN*, *COL6A1*, *COL6A2*) and enhanced inflammatory gene signature (*MMP1*, *MMP3*, *IGFB3*, *IL6*, *TNFAIP6*) in fibroblasts from cultured skin. These results pointed to a significant shift in transcriptomes of stromal cell populations between fresh and cultured skin scRNA-seq, however these data could be confounded by biological differences between enrolled patients (unaffected skin from patients undergoing skin surgery versus SSc skin).

To further understand the potential effects of technical (protocol) and inter-donor biological variability on these expression changes, we interrogated the expression of the identified differentially expressed genes (Figure 6D) in our integrated scRNA-seq skin atlas (Figure 5). Since fibroblasts showed the most profound changes in gene expression under cultured conditions, we focused on the subset of differentially expressed fibroblast genes (Figure 6D). This analysis showed a certain variation in gene expression within and between the studies (Supplementary Figure S10), however none of the studies exhibited high levels of MMP1, MMP3, IGFBP3 or IL6 in fibroblast clusters as detected in cultured skin explants (Figure 6D). This analysis suggested that culturing conditions or SSc diagnosis could contribute to the differential expression of these fibroblast genes in cultured skin.

## Discussion

Skin tissue digestion protocols have significant impact on the yield and quality of isolated cells, as well as their gene expression (Botting et al., 2017). Multiple studies (Der et al., 2019; Devitt et al., 2019; Gaydosik et al., 2019; He et al., 2020; Kim et al., 2020; Mirizio et al., 2020; Tabib et al., 2021) have employed different dissociation protocols, resulting in varying number and survival of isolated cells, with potential effect on cell gene expression profiles and capture of different skin cell populations. Currently, there is an unmet need for a standardized and reproducible protocol for skin dissociation. Here, we described an optimized protocol employing a sequential combined enzymatic (dispase II, collagenase IV, trypsin) and gentle mechanical (shaking, stirring) cell dissociation approach that resulted in a large number of isolated highly viable skin cells. High number of viable cells in our protocol efficiently contributed to representative capture of human skin cell heterogeneity and enabled a robust detection of transcriptomic skin cell states. Additionally, enabling the isolation of viable cells in high numbers from small-sized fresh and cultured human punch skin biopsies, our protocol demonstrated a broad research applicability and may be used for *ex vivo* skin explant experimentation and research of early skin disease stages where tissue material is limited. Reduced reagent costs for tissue digestion present an important characteristic of our protocol compared to commercially available protocols.

Analyzing our fresh skin scRNA-seq datasets independently and integrating them with published skin datasets showed that our protocol recapitulates broad cell composition of adult human skin by scRNA-seq. Notably, we efficiently captured also rare skin cell populations, such as mast cells, despite sequencing a smaller total number of isolated skin cells (He et al., 2020; Solé-Boldo et al., 2020; Tabib et al., 2021). In accordance with other studies (Solé-Boldo et al., 2020; Tabib et al., 2021), fibroblasts represented the most abundant skin cell population also in our fresh skin dataset. We were able to discriminate reticular and papillary fibroblast subsets reflecting their spatial positioning within anatomic skin compartments, which has been demonstrated in some, but not all studies (Vorstandlechner et al., 2020; Ascensión et al., 2021). Different fibroblast subsets have been identified in different studies. Investigating skin aging, Solé-Boldo et al*.* (2020), identified four main young fibroblast populations composed of secretory reticular (*WISP2*, *SLPI*, *MFAP5*, *TSPAN8*), secretory papillary (*APCDD1*, *ID1*, *WIF1*, *COL18A1*, *PTGDS*), mesenchymal (*ASPN*, *POSTN*, *GPC3*, *TNN*, *SFRP1*) and inflammatory (*CCL19*, *APOE*, *CXLC2*, *CXCL3*, *EFEMP1*) fibroblast subsets. Tabib et al. (2021) described two main fibroblasts subtypes in healthy skin, including ECM-related SFRP2+/DPP4+ cells and inflammatory APOE/FMO1+ population, with no recognizable resemblance to papillary or reticular fibroblasts. *SFPR2* and *FMO1* markers showed low-expression in our dataset and were not reliable separators of fibroblast clusters neither in our, nor in the Solé-Boldo study. A study by Philippeous et al. (2018) proposed four fibroblast subpopulations including two clusters with distinctive expression profiles of papillary (*COL6A5*, *COL23A1*, *HSPB3*) and reticular cells (*CD26*, *MFAP5*, *PRG4*). Our subclustering of fibroblasts demonstrated some overlap with secretory papillary and secretory reticular fibroblasts defined by Solé-Boldo et al*.* (2020) and group 3 papillary and group 4 reticular fibroblasts from Philippeous et al. (2018) data. Additionally, our inflammatory CCL19+ C3+ cluster 2 fibroblasts [with low expression of collagen genes (*COL1A1*, *COL1A2*, *COL6A1*, *COL6A2*)] were also observed in previous studies. Specifically, Solé-Boldo et al. described a pro-inflammatory CCL19+, APOE+ fibroblast cluster [defined by reticular gene signature, low expression of collagen genes and being located predominantly near the vasculature (Solé-Boldo et al., 2020)], while Tabib et al. described pro-inflammatory CCL19+, C7+, APOE^high^ perivascular fibroblasts (Tabib et al., 2021).

Keratinocytes in our dataset exhibited similar subset diversity as described in the neonatal foreskin epidermis by Wang et al. (2020) including basal, spinous, and granular keratinocyte subpopulations. Furthermore, we identified a transitional KRT1^med^KRT15^med^ keratinocyte subset pointing towards the continuum of keratinocyte differentiation states in human skin. In addition to basal and suprabasal keratinocytes, He et al. (2020) described the inner/outer root sheath, sebaceous gland and ion channel (ATP-ase and channel gap) keratinocyte signatures in healthy and atopic dermatitis skin, which were not clearly distinguished in our analysis.

Hughes et al. (2020) described several myeloid populations from different inflammatory skin diseases, and a signature of one of the macrophage clusters, found in healthy skin, overlapped with macrophage cluster genes in our study. Whereas Hughes et al. identified five distinct clusters of conventional and dermal DCs in inflamed and healthy skin, this DC diversity could not be clearly delineated in our analysis possibly due to limited myeloid cell counts and small sampling size.

In a proof-of-concept experiment with cultured skin explants, we demonstrated that our skin dissociation protocol facilitates the generation of high quality scRNA-seq data not only from fresh, but also from cultured skin obtained from clinically relevant small punch skin biopsies from patients with SSc. Notably, our integrative analysis of cultured and fresh skin scRNAseq data revealed striking alterations in skin cell transcriptomes under cultured conditions, primarily affecting the stromal skin cell compartment. In tissues, cell identity and subspecialization are guided by anatomical body localization (Chang et al., 2002), subtissular niche, local microenvironmental signals, interactions with neighboring cells and homeostatic versus disease states. Tissue microenvironment changes under cell culture conditions which could induce transcriptional changes in cultured tissue explants. When cultured, skin fibroblasts (Walmsley et al., 2015; Philippeos et al., 2018; Korosec et al., 2019) but also other cell types (Kim et al., 2018; Wei et al., 2020) lose the expression of subset-specific identity markers and tissue-linked cell subset heterogeneity. Despite marker loss under culture conditions, different skin fibroblast subsets could regain their original functions when introduced in decellularized dermis.

Our results raise an important question about potential transcriptomic shifts across principal resident skin cell populations happening under *ex vivo* culturing of the human skin. These findings suggest that tissue culturing and preservation conditions should be carefully tested when using, e.g., scRNA-seq as a readout in experiments in *ex vivo* cultured human skin. The comparison of scRNA-seq data from cultured and freshly dissociated skin in our proof-of-concept experimental setup has been confounded by different biological backgrounds of enrolled study subjects (SSc vs. nonaffected skin from skin surgery patients). Specifically, our primary aim in this study was testing the feasibility of our optimized protocol for cell dissociation and scRNA-seq studies on cultured skin explants derived from clinically relevant patient material. The access to valuable SSc biopsies has been limited and we did not use SSc samples for protocol establishment and optimization. Future studies on paired fresh and cultured skin fragments from the same individuals should further clarify culture-induced transcriptomic shifts in tissue-populating cell types to understand the experimental feasibility and limitations of human skin cell models.

## Conclusion

Preparation of high-quality single cell suspensions represents a crucial but often neglected step in scRNA-seq data generation, representing a significant drawback especially for difficult to digest tissues, such as skin. Here, we describe a new protocol optimized for digestion of small-sized, fresh, and cultured skin biopsies, resulting in a high yield of viable skin cells. Using scRNA-seq analysis we confirm that our protocol enables the capture of all previously described skin cell types including the rare skin cell populations utilizing a limited number of skin samples. Furthermore, our analysis uncovers potential transcriptomic shifts in *ex vivo* cultured skin, pointing to cautious interpretation of scRNA-seq data from different tissue culturing and storage conditions. Overall, our new protocol increases high quality viable cell yields and could contribute to diminishing the bias in scRNA-seq analyses with improved representation of cell heterogeneity in human skin.

## Data Availability

The datasets presented in this study can be found in online repositories. The names of the repository/repositories and accession number(s) can be found below: ArrayExpress from the EMBL-EBI. The data has the accession number E-MTAB-11509 (https://www.ebi.ac.uk/arrayexpress/experiments/E-MTAB-11509).
