# Supplementary material for: An Optimized Tissue Dissociation Protocol for Single-Cell RNA Sequencing Analysis of Fresh and Cultured Human Skin Biopsies
Source: Front Cell Dev Biol. 2022 Apr 28;10:872688. doi: 10.3389/fcell.2022.872688 (PMC9096112; doi:10.3389/fcell.2022.872688)
Supplement: Supplementary file 1 [file Table1.DOCX]

**Supplementary Table I.** Clinical characteristics of healthy skin donors and patients with systemic sclerosis that provided skin biopsies for cultured experiments.

| Subject | **HC 1** | **HC 2** | **SSc 1** | **SSc 2** | **SSc 3** | **SSc 4** |
| --- | --- | --- | --- | --- | --- | --- |
| Sample name | Fresh_S1 | Fresh_S2 | Culture_S1 | Culture_S2 | Culture_S4 | Culture_S5 |
| Biopsy site | breast | lower leg | left forearm | left forearm | left forearm | left forearm |
| Disease | Non-diseased skin | Non-diseased skin | SSc | SSc | SSc | SSc |
| Gender | female | men | male | male | male | female |
| Age | 46 | 79 | 59 | 55 | 55 | 83 |
| Operation procedure | breast reduction surgery (ptosis of the breast) | cutaneous squamous cell carcinoma removal | NA | NA | NA | NA |

**Supplementary Table II.** Publicly available studies utilizing different skin tissue digestions protocols for scRNA-seq applications. HC: healty control; SSc: systemic sclerosis; NA: non-applicable

| Paper | Skin punch biopsy size (mm) | fresh/frozen | Patient (numbers) | Dissociation protocol | Isolated cell numbers | Viability |
| --- | --- | --- | --- | --- | --- | --- |
| He et al.[1] | 2.5-5 | frozen | 5 atopic dermatitis, 7 HC | Liberase+trypsin | NA | 43% to 77%. |
| Devitt [2] | NA | fresh | 1 HC | dispase + trypsin | 12,000 | 80% |
| Mirizio [3] | 3 | fresh | 3 SSc | Milteny MACS | 1,274 – 3,846 | 70–80% |
|  | 3 | frozen | 3 SSc | Milteny MACS | 1,707-2,576 | 60–75% |
| Gaydosik [4] | 3 | NA | 27 SSc | Milteny MACS | 870-3,179 | NA |
|  | 3 | NA | 10 HC | Milteny MACS | 1,035-3,145 | NA |
| Tabib [5]. | 3 | NA | 10 HC | Milteny MACS | mean 2,821 | NA |
|  | 3 | NA | 12 SSc | Milteny MACS | mean 3,082 | NA |

| Dataset | #Donors | Age | Sex (M-male, F-female) | Anatomic area | Cell number total | Fresh/frozen | Time from harvest to processing | Dissociation protocol | Cell viability | Cell type enrichment | Platform and chemistry (10x Genomics) |  |
| --- | --- | --- | --- | --- | --- | --- | --- | --- | --- | --- | --- | --- |
| Solé-Boldo [6] | 5 | 25, 27, 53, 70, 69 | M | Inguinoiliac  (sun  protected) | 15,457 | fresh | <1 h | Whole-skin dissociation kit + gentleMACS (Miltenyi Biotec) | NA | Magnetic  isolation of  live cells | Chromium Single Cell  3’ version 2 |  |
| He etl al. [1] | 7 | 38-82 | 4 F, 3 M | Unspecified Extremities | 22,220 | frozen | Punch biopsies  stored at ‒80°C for  4‒5 months | Liberase TL (2X) Trypsin, | 43‒77% viable cells | No | Chromium Single Cell  3’ version 2 |  |
| Tabib et al. [5] | 10 | 23, 24, 54, 62, 63, 66, 4X NA | 4 F, 6 M | Dorsal midforearm | NA | fresh | Unspecified | Whole-skin dissociation  kit gentleMACS  (Miltenyi Biotec) | NA | No | Chromium Single Cell  3’ version 1 and 2 |  |

**Supplementary Table III.** Publicly available healthy skin datasets used for integration analysis.

[1] H. He *et al.*, "Single-cell transcriptome analysis of human skin identifies novel fibroblast subpopulation and enrichment of immune subsets in atopic dermatitis," (in eng), *J Allergy Clin Immunol,* vol. 145, no. 6, pp. 1615-1628, 06 2020, doi: 10.1016/j.jaci.2020.01.042.

[2] K. Devitt *et al.*, "Single-cell RNA sequencing reveals cell type-specific HPV expression in hyperplastic skin lesions," (in eng), *Virology,* vol. 537, pp. 14-19, 11 2019, doi: 10.1016/j.virol.2019.08.007.

[3] E. Mirizio *et al.*, "Single-cell transcriptome conservation in a comparative analysis of fresh and cryopreserved human skin tissue: pilot in localized scleroderma," (in eng), *Arthritis Res Ther,* vol. 22, no. 1, p. 263, 11 09 2020, doi: 10.1186/s13075-020-02343-4.

[4] A. M. Gaydosik, T. Tabib, R. Domsic, D. Khanna, R. Lafyatis, and P. Fuschiotti, "Single-cell transcriptome analysis identifies skin-specific T-cell responses in systemic sclerosis," (in eng), *Ann Rheum Dis,* vol. 80, no. 11, pp. 1453-1460, 11 2021, doi: 10.1136/annrheumdis-2021-220209.

[5] T. Tabib *et al.*, "Myofibroblast transcriptome indicates SFRP2," (in eng), *Nat Commun,* vol. 12, no. 1, p. 4384, 07 19 2021, doi: 10.1038/s41467-021-24607-6.

[6] L. Solé-Boldo *et al.*, "Single-cell transcriptomes of the human skin reveal age-related loss of fibroblast priming," (in eng), *Commun Biol,* vol. 3, no. 1, p. 188, 04 23 2020, doi: 10.1038/s42003-020-0922-4.
