# Supplementary material for: An Optimized Tissue Dissociation Protocol for Single-Cell RNA Sequencing Analysis of Fresh and Cultured Human Skin Biopsies
Source: Front Cell Dev Biol. 2022 Apr 28;10:872688. doi: 10.3389/fcell.2022.872688 (PMC9096112; doi:10.3389/fcell.2022.872688)

## Slide 1
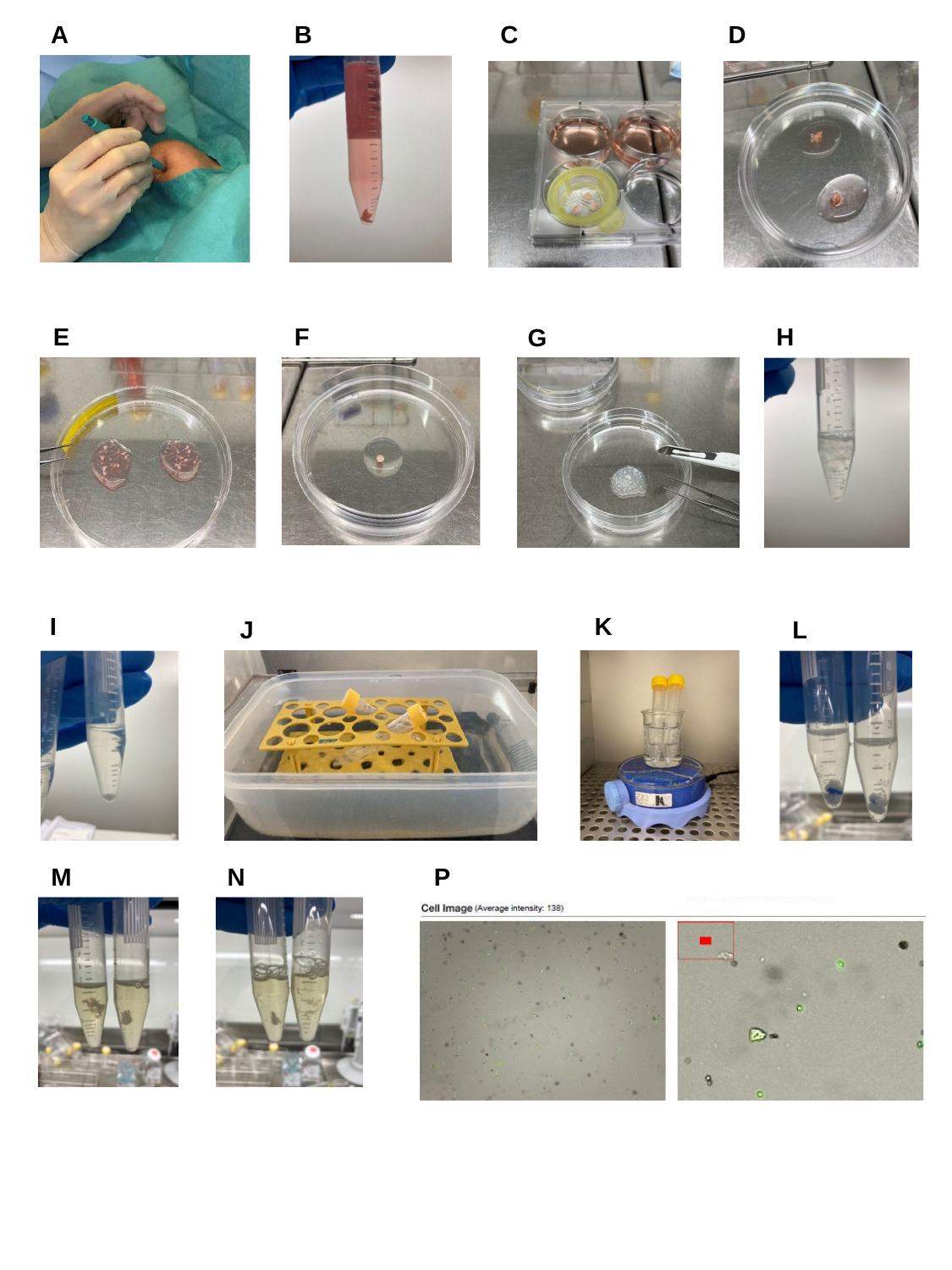

D
A
B
C
H
E
F
G
K
I
J
L
M
N
P

## Slide 2
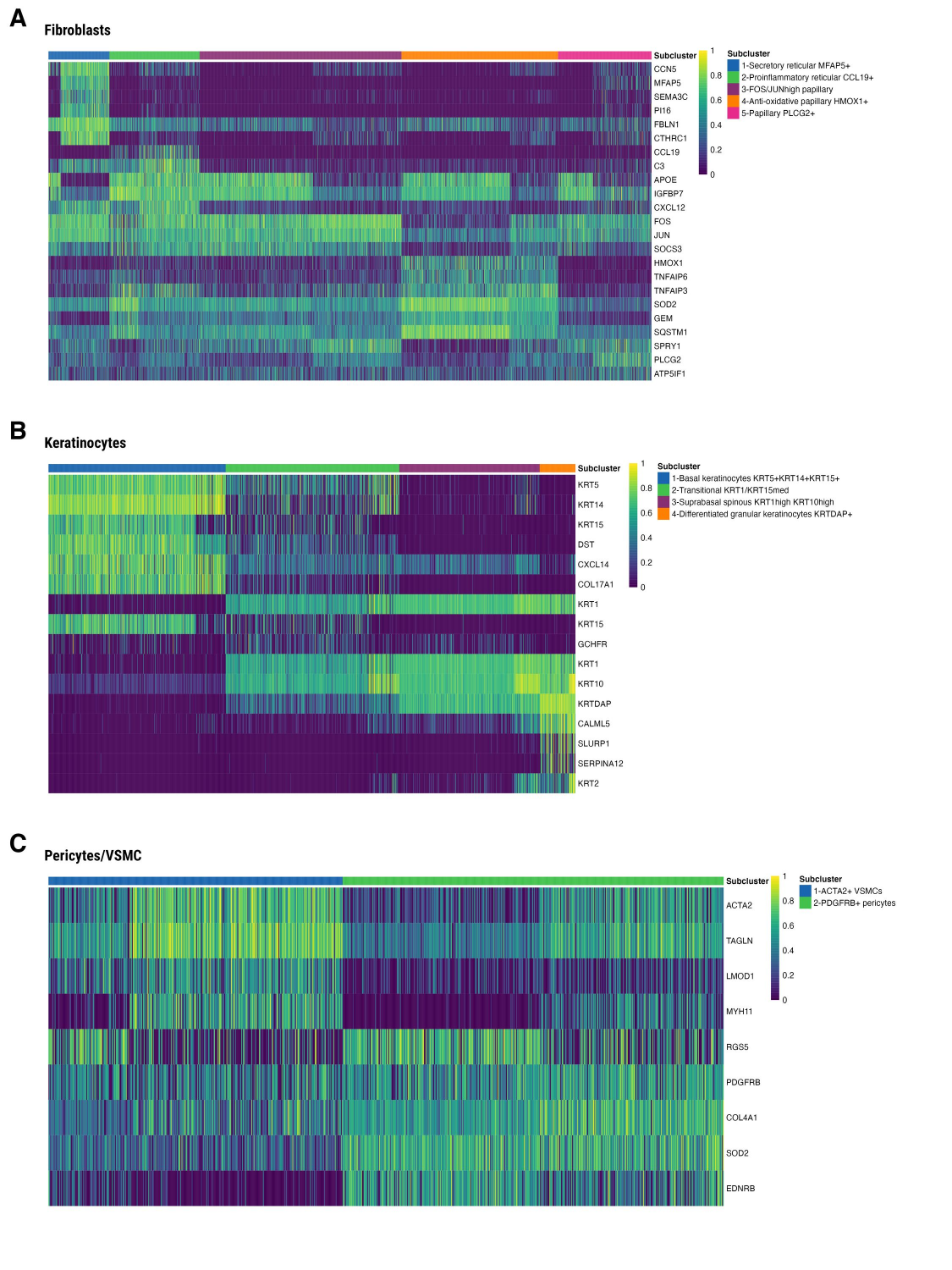

## Slide 3
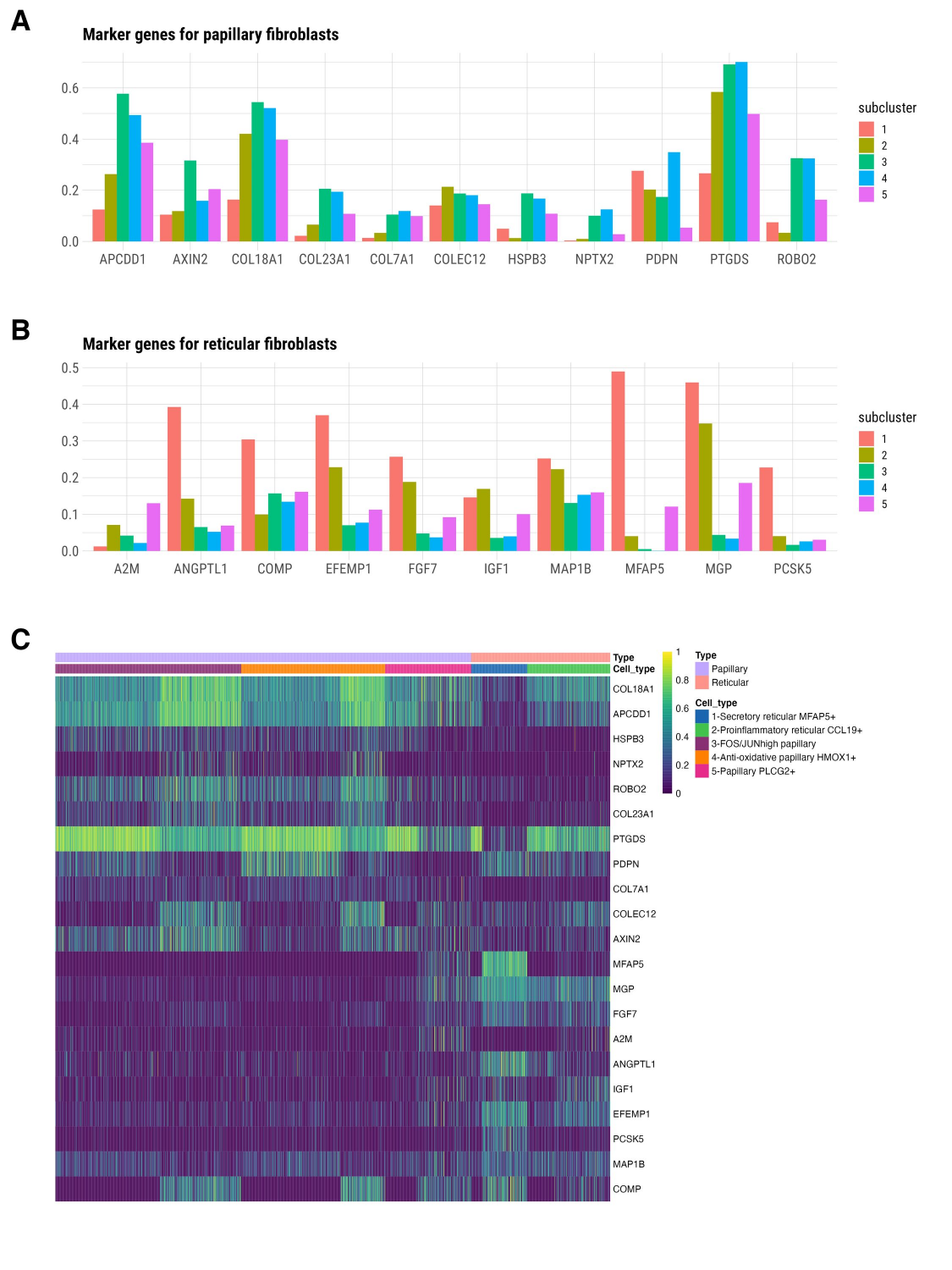

## Slide 4
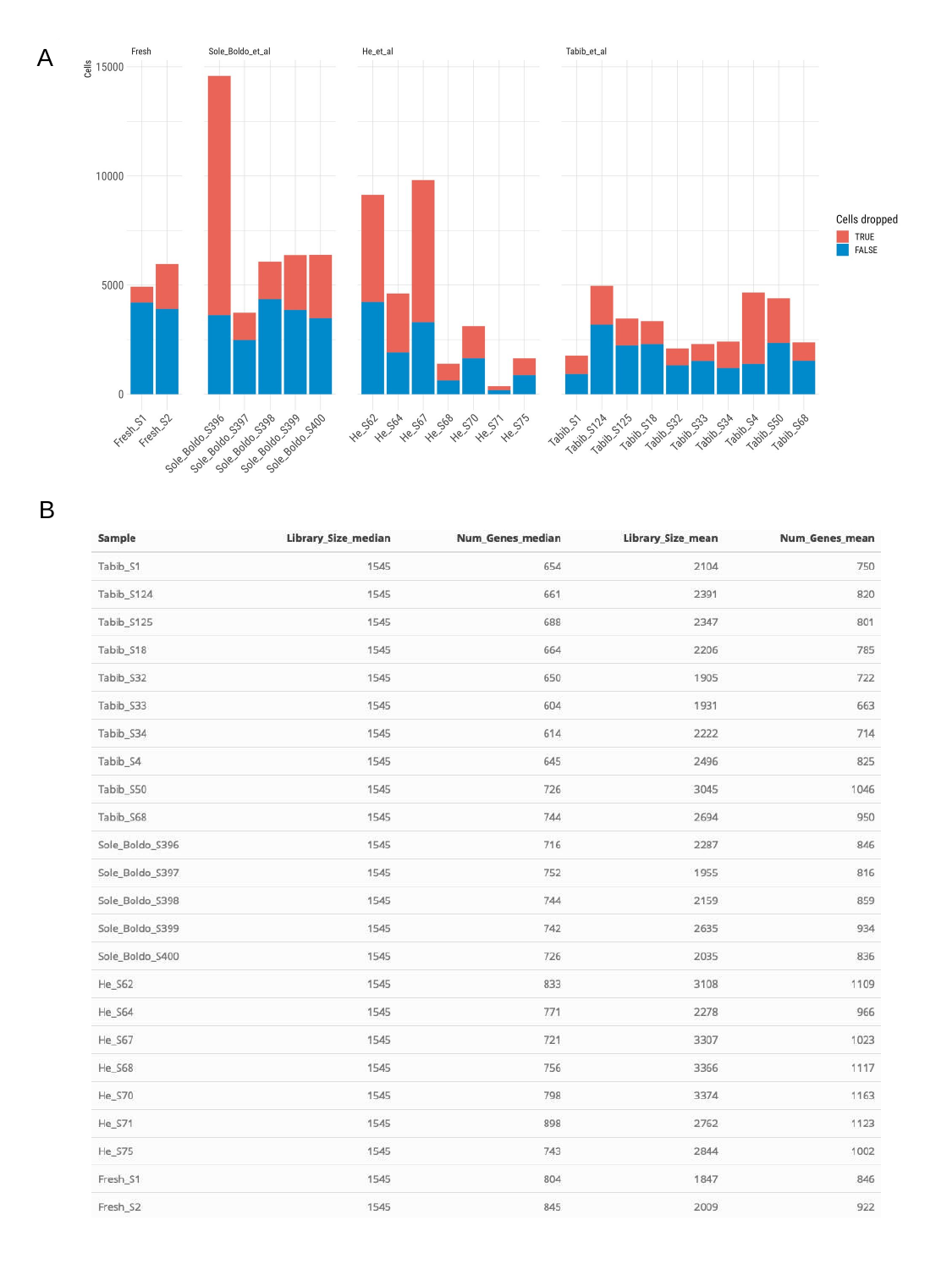

A
B

## Slide 5
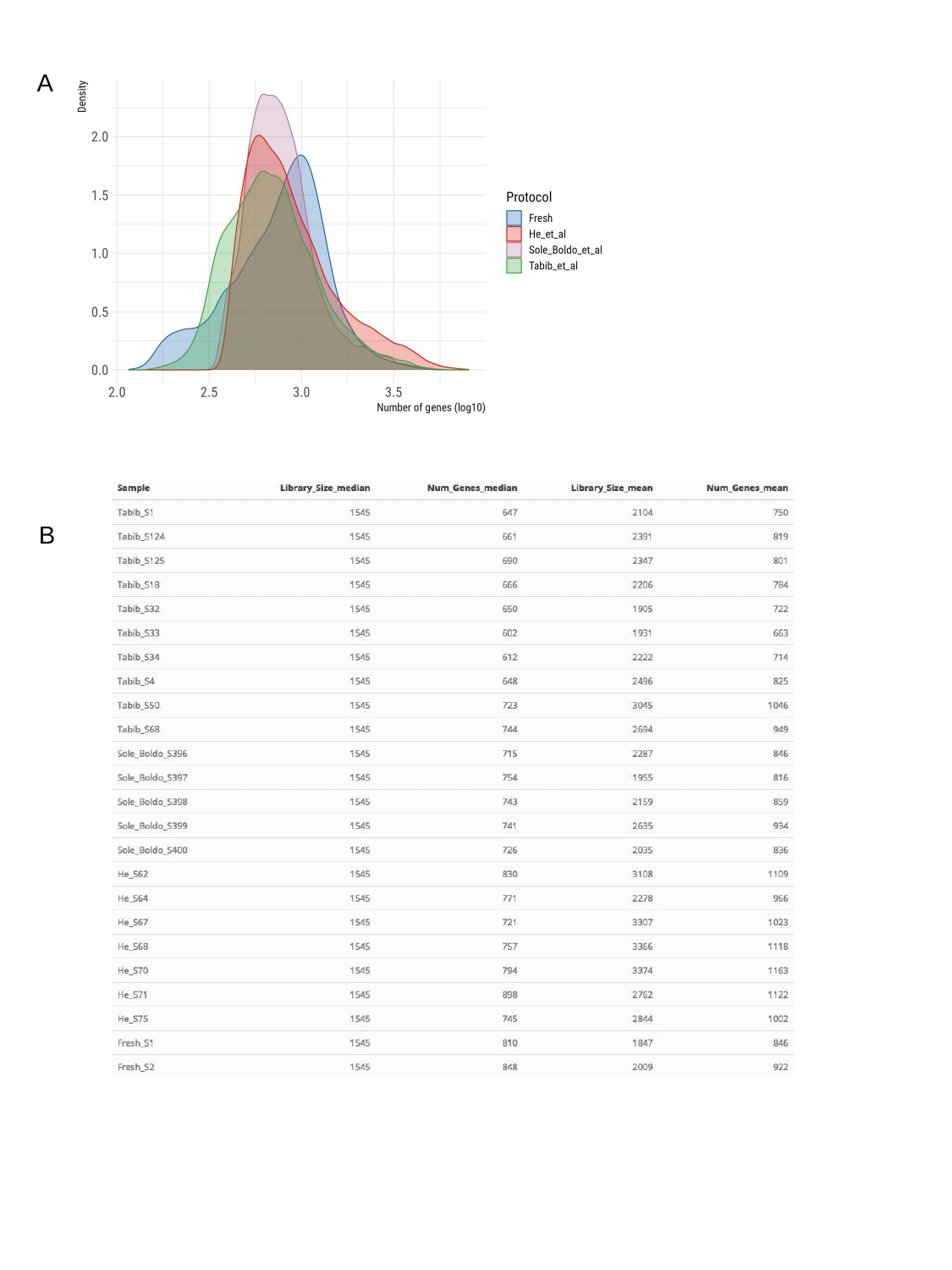

A
B

## Slide 6
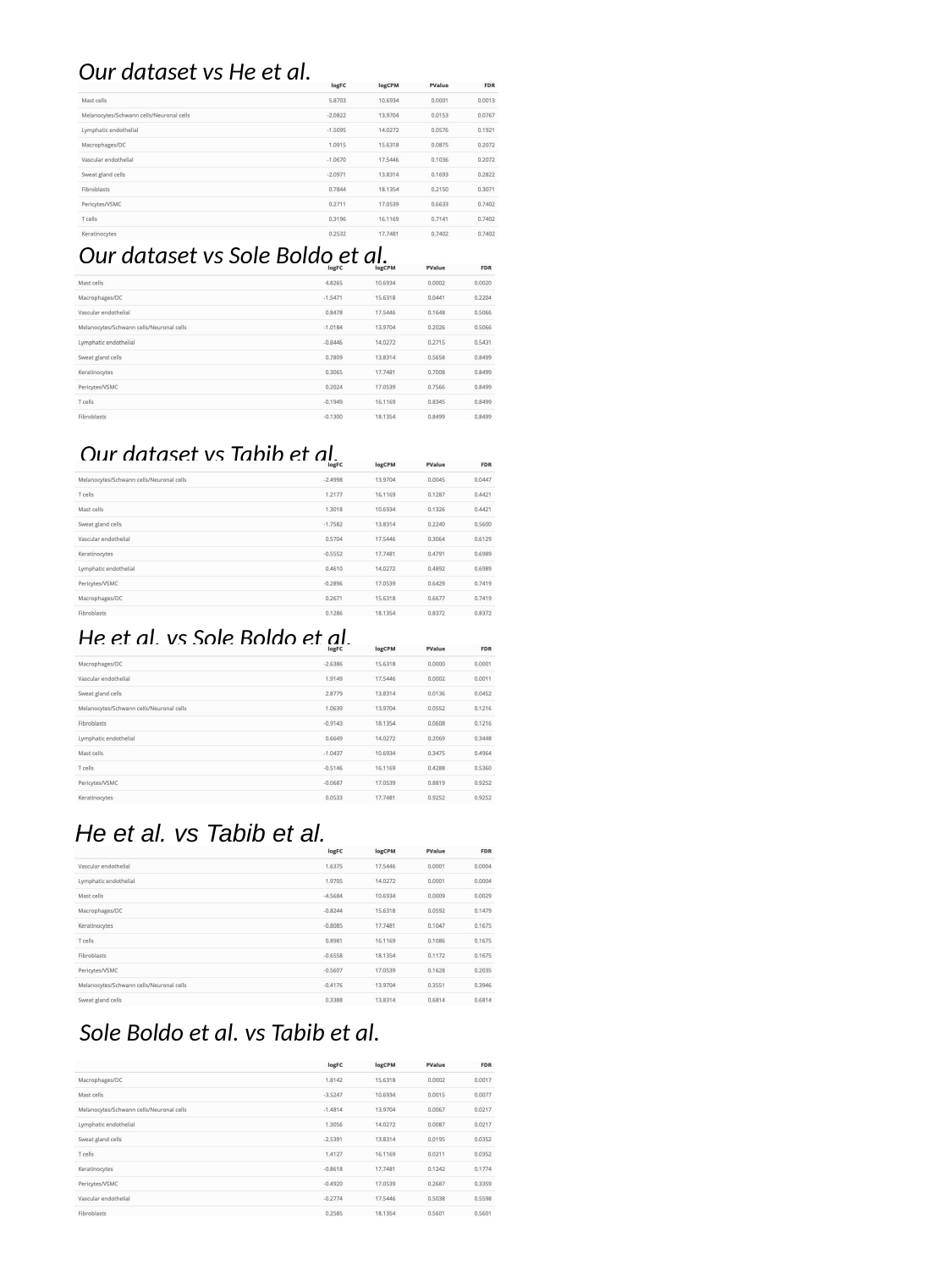

Our dataset vs He et al.
Our dataset vs Sole Boldo et al.
Our dataset vs Tabib et al.
He et al. vs Sole Boldo et al.
He et al. vs Tabib et al.
Sole Boldo et al. vs Tabib et al.

## Slide 7
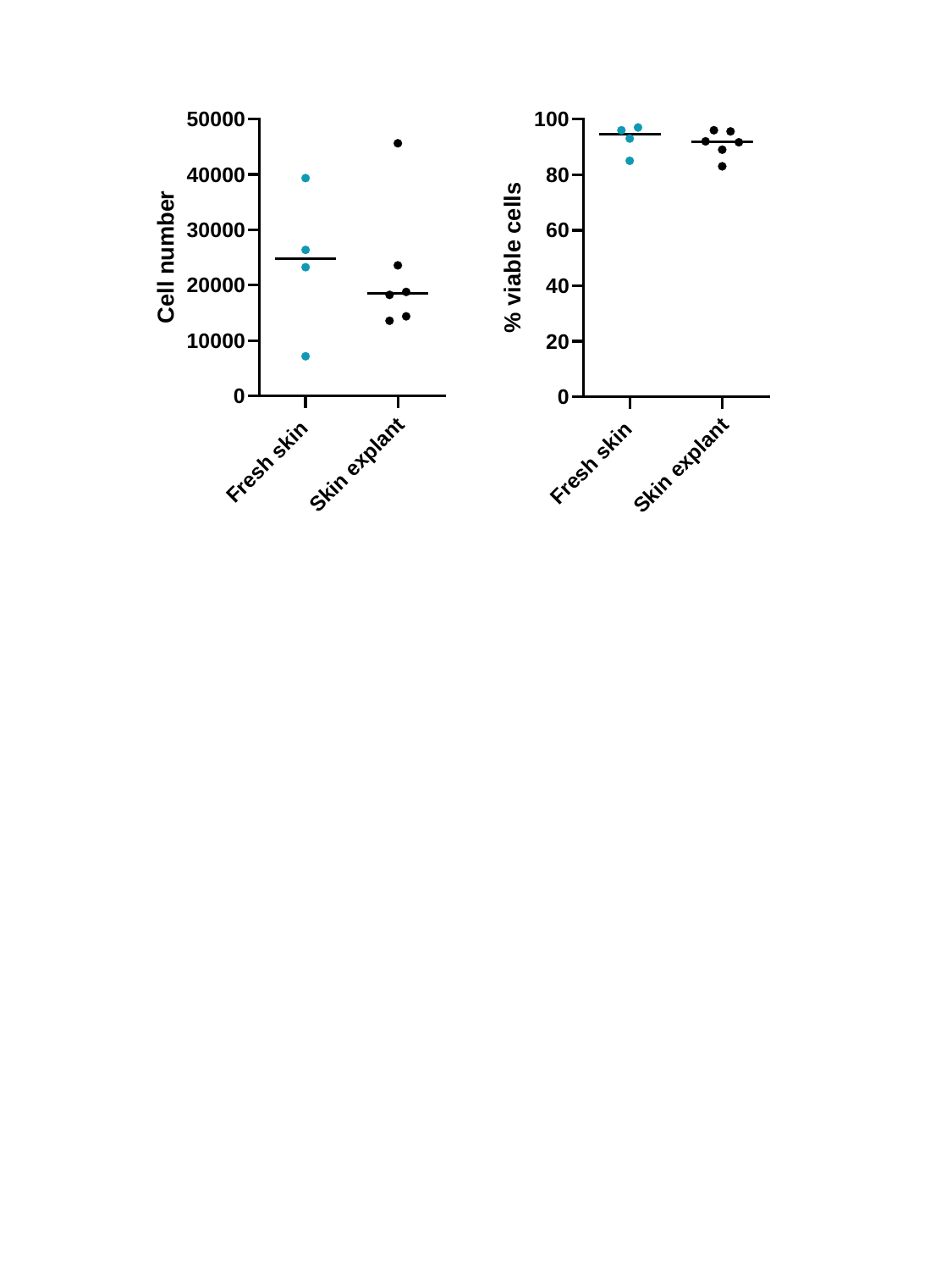

## Slide 8
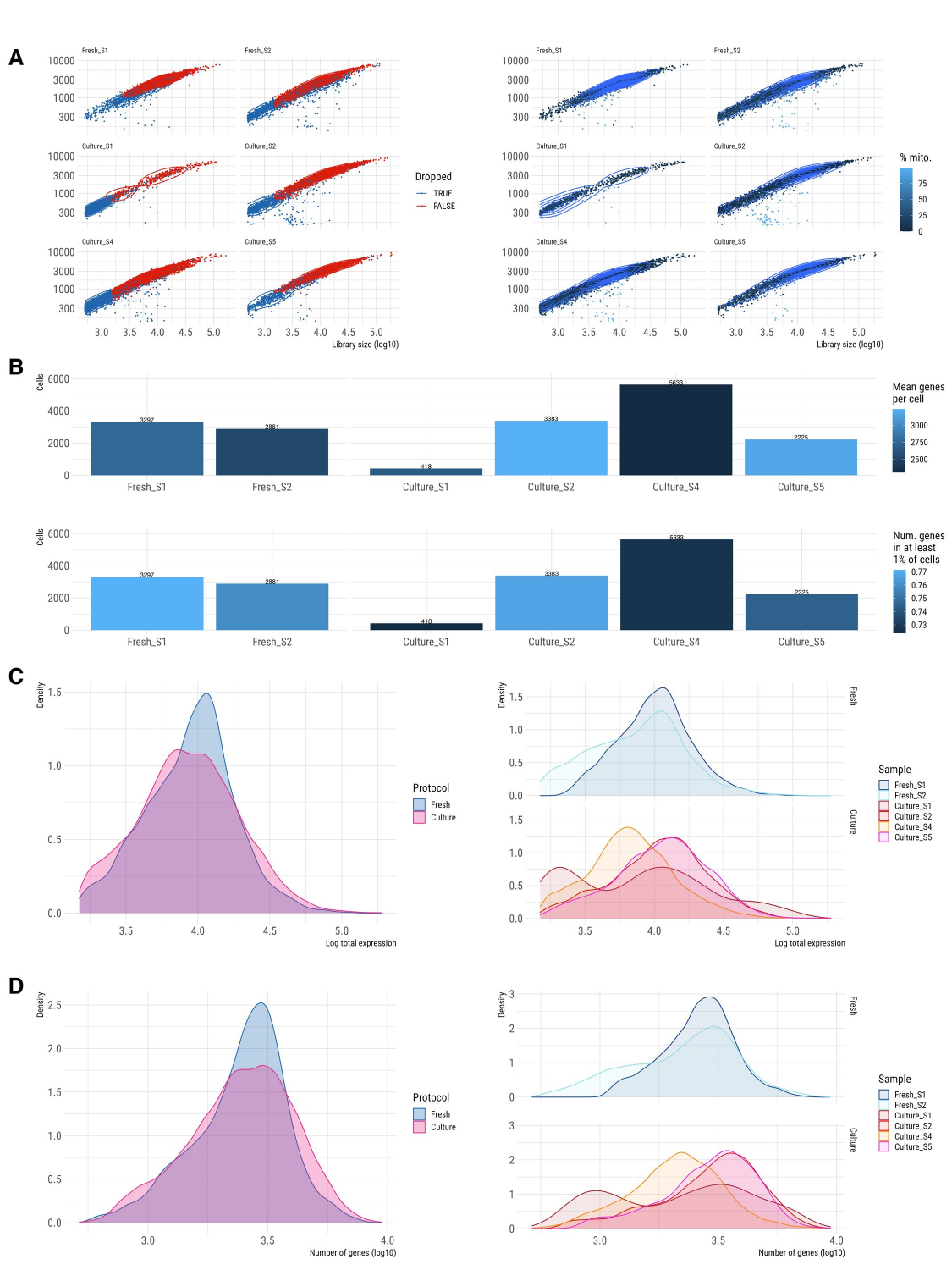

## Slide 9
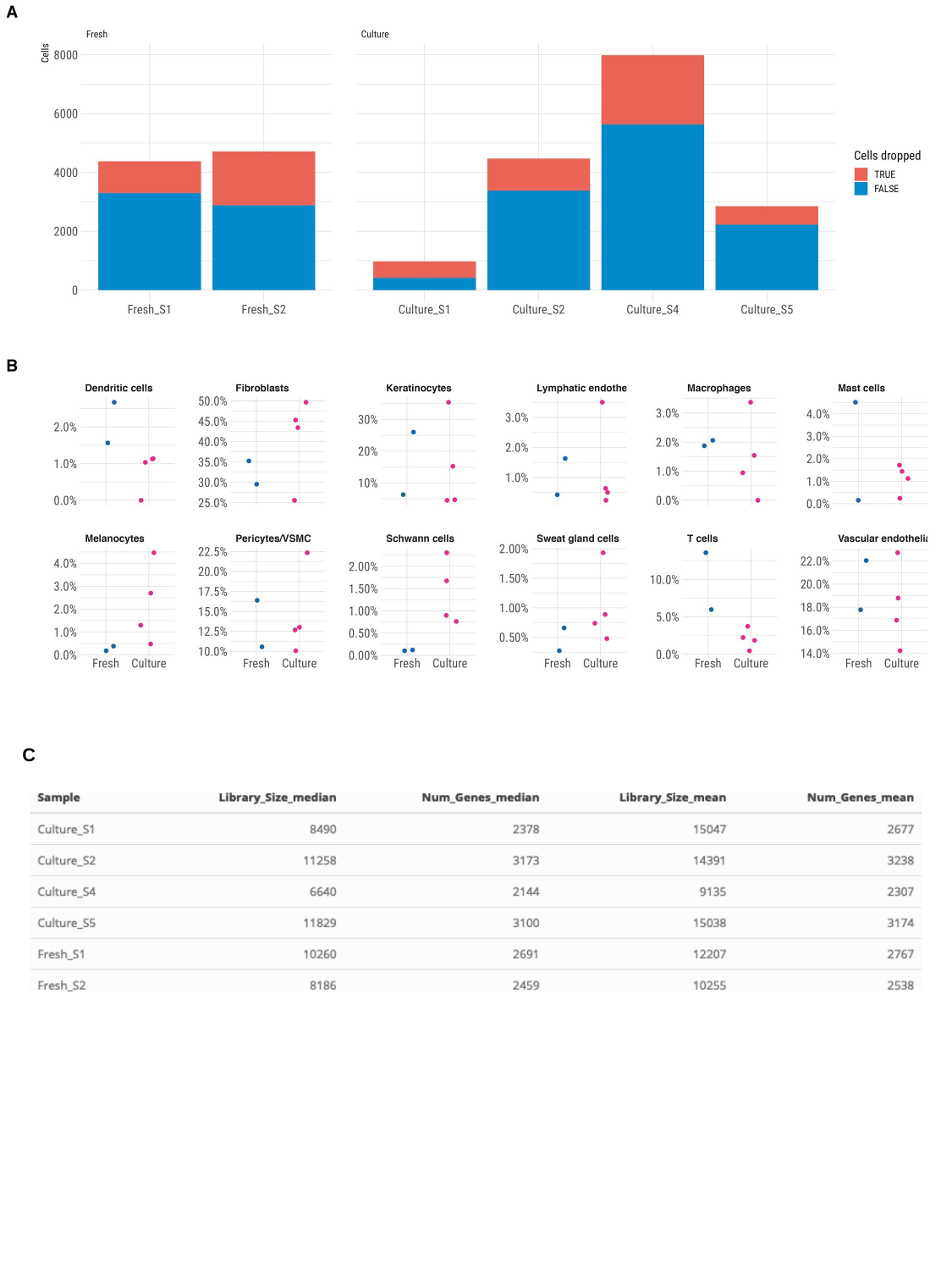

C

## Slide 10
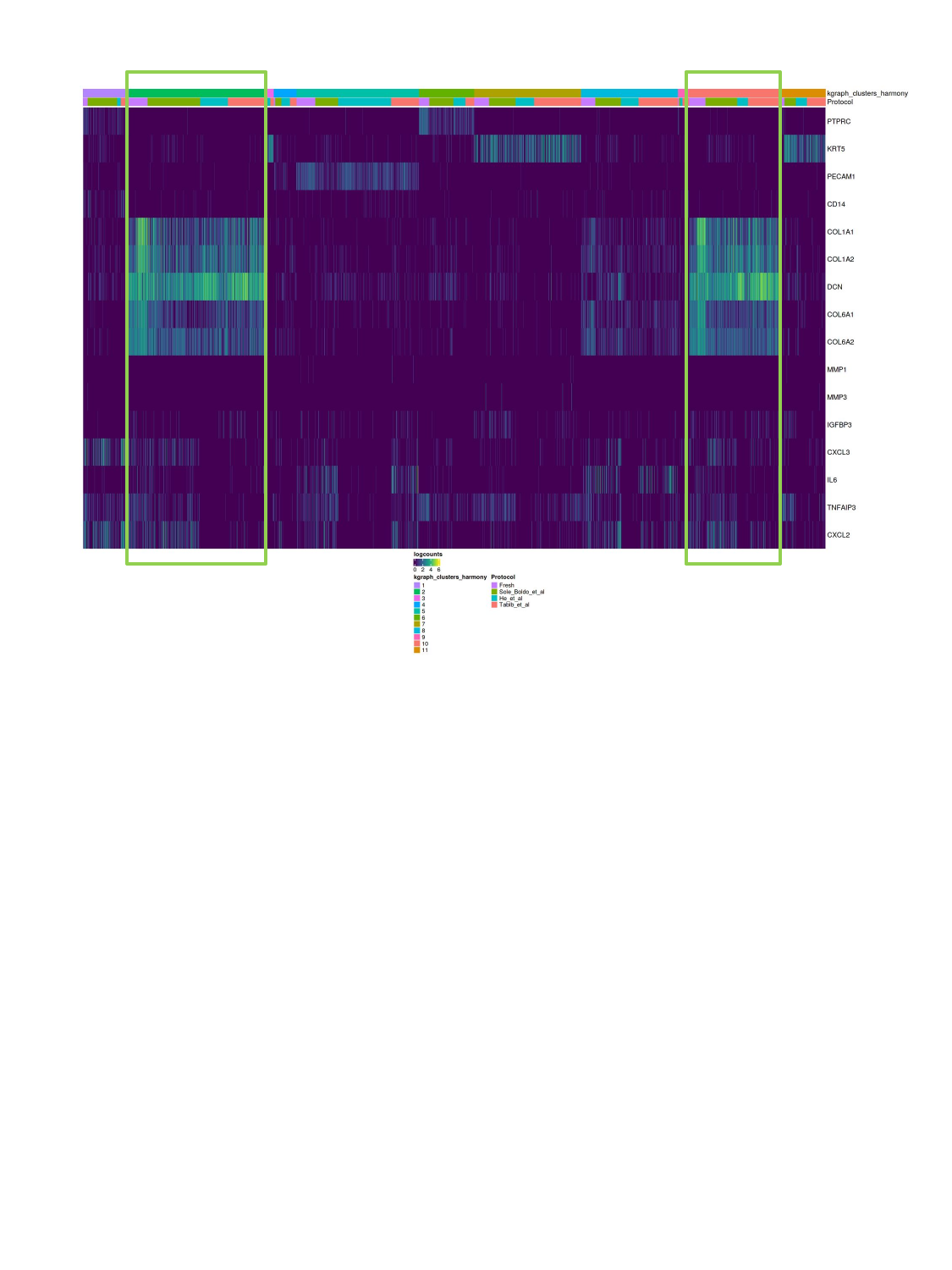

Supplement: Supplementary file 2 [file Presentation1.PPTX]
